# The effects of dietary nitrate supplementation on endurance exercise performance and cardiorespiratory measures in healthy adults: a systematic review and meta-analysis

**DOI:** 10.1186/s12970-021-00450-4

**Published:** 2021-07-09

**Authors:** Chloe Gao, Saurabh Gupta, Taranah Adli, Winston Hou, Reid Coolsaet, Abigail Hayes, Kevin Kim, Arjun Pandey, Jacob Gordon, Gurneet Chahil, Emilie P. Belley-Cote, Richard P. Whitlock

**Affiliations:** 1 Faculty of Medicine, University of British Columbia, Vancouver, British Columbia Canada; 2 Department of Surgery, McMaster University, Hamilton, Ontario Canada; 3 Faculty of Biomedical Sciences, University of Western Ontario, London, Ontario Canada; 4 Faculty of Health Sciences, McMaster University, Hamilton, Ontario Canada; 5 University of Guelph, Guelph, Ontario Canada; 6 Queen’s University, Kingston, Ontario Canada; 7 Michael G. DeGroote School of Medicine, McMaster University, Hamilton, Ontario Canada; 8 Department of Health Research Methods, Evidence and Impact, McMaster University, Hamilton, Ontario Canada; 9 Population Health Research Institute, Hamilton, Ontario Canada; 10 Saint James School of Medicine, Park Ridge, USA; 11 Department of Medicine, McMaster University, Hamilton, Ontario Canada; 12 David Braley Cardiac, Vascular and Stroke Research Institute, 237 Barton St. E., Hamilton, Ontario L8L 2X2 Canada

**Keywords:** Nitrate supplementation, Endurance exercise, Systematic review and meta-analysis

## Abstract

**Background:**

Nitrate supplementation is thought to improve performance in endurance sports.

**Objective:**

To meta-analyze studies evaluating the effect of nitrate supplementation on endurance sports performance among adults.

**Data sources:**

We searched the Cochrane Central Register of Controlled Trials (CENTRAL), MEDLINE, EMBASE, Web of Science and CINAHL without language restrictions.

**Methods:**

We included studies that: 1) compared nitrate supplementation with placebo; 2) enrolled adults engaging in an endurance-based activity; and 3) reported a performance measure or surrogate physiologic outcome. We evaluated risk of bias using the Cochrane Collaboration tool and pooled data with a random-effects model. We used the Grading of Recommendations Assessment, Development and Evaluation (GRADE) approach to evaluate confidence in estimates.

**Results:**

We included 73 studies (*n* = 1061). Nitrate supplementation improved power output (MD 4.6 watts, *P* < 0.0001), time to exhaustion (MD 25.3 s, *P* < 0.00001), and distance travelled (MD 163.7 m, *P* = 0.03). We found no significant difference on perceived exertion, time trial performance and work done. Nitrate supplementation decreased VO_2_ (MD − 0.04 L/min, *P* < 0.00001) but had no significant effect on VO_2max_ or blood lactate levels.

**Conclusion:**

The available evidence suggests that dietary nitrate supplementation benefits performance-related outcomes for endurance sports.

**Supplementary Information:**

The online version contains supplementary material available at 10.1186/s12970-021-00450-4.

## Strengths and limitations of this study

- Systematic review and meta-analysis of randomized studies with a comprehensive search strategy and pre-specified protocol
- Rigorous methods and systematic evaluation of randomized data
- Insufficient details on randomization procedures in study reports, leading to unclear risk of bias
- Heterogeneity in study methodologies and outcome reporting

## Background

**Fig. 1:**
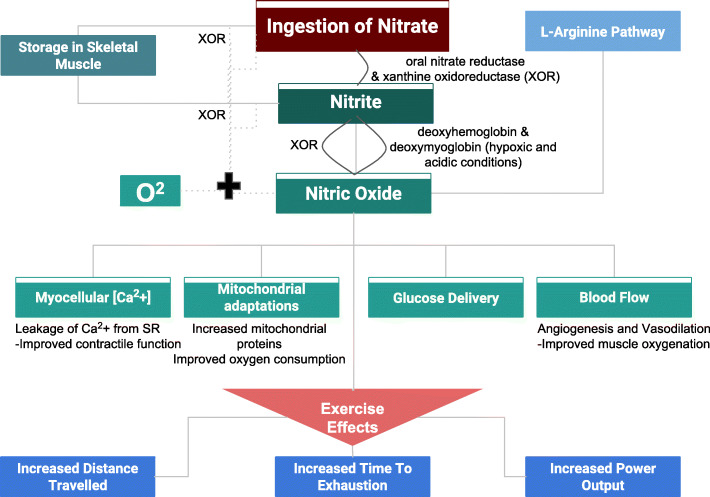
Possible mechanisms of effect of nitrate on athletic performance

Endurance capacity is an important component of physical fitness that relates to the ability of the circulatory and respiratory systems to support sustained physical activity [1]. The performance of athletes training and competing in sports such as distance running, triathlons, swimming, biking and rowing depends on their endurance capacity [2, 3]. Different macronutrients and micronutrients have been used as ergogenic aids to potentially improve performance [4]. Specifically, nitrates are thought to potentially improve athletic performance. Beetroot juice, pomegranate extract and green leafy vegetables such as collard greens, lettuce, and spinach constitute substantial sources of dietary nitrate [5]. While the exact mechanism underlying the ergogenic benefits of nitrate supplementation has not yet been established, it has been proposed that dietary nitrate, once ingested, is reduced to nitric oxide (NO). Classically, NO was thought to be generated by oxidation of L-arginine, resulting in endogenous production of nitrate (NO3-) and nitrite (NO2-). A vasodilator, it is believed to influence muscle function by modulating skeletal muscle function through its role in blood flow regulation, contractility, glucose and calcium homeostasis, and mitochondrial biogenesis [5]. Increased levels of NO in tissues and peripheral circulation may lead to improved oxygen transport and uptake in muscles during exercise [3, 6]. Figure 1 gives details on the potential mechanisms that nitrate supplementation can, eventually, result in improved physical performance.

While animal studies (in dogs, cats and horses) have demonstrated that reduction in endogenous NO production increases oxygen consumption (VO2), controversy remains in human performance. In fact, recent research suggesting nitrate supplementation may have performance benefits has resulted in its increased popularity among individuals attempting to improve their athletic performance [7]. However, the results of primary investigations examining nitrate supplementation have been inconsistent, with some studies suggesting a benefit ([8–11]) and others no effect ([12–15]). In reviewing these studies, along with input from elite athlete (co-author, RC), we decided to focus on power output, time to exhaustion, and VO2, among others as metrics of athletic performance.

This systematic review and meta-analysis aimed to summarize the placebo-controlled trials evaluating dietary nitrate supplementation’s effect on endurance exercise performance.

## Methods

### Identification of studies

We searched the Cochrane Central Register of Controlled Trials (CENTRAL), MEDLINE, EMBASE, Web of Science and CINAHL from inception to February 2021 without language restrictions (Search strategy – Supplementary Table 1).

### Study inclusion and selection

We included comparative studies that examined the effect of dietary nitrate supplementation on endurance activities. We performed title and abstract screening independently and in duplicate using the Covidence online software (Veritas Health Innovation, Melbourne, Australia). If either reviewer deemed a study relevant, we retrieved it for full-text review. We resolved disagreements between reviewers regarding eligibility through discussion or third-party arbitration. Eligible studies had to meet the following criteria:

The population of interest was healthy adults (over the age of 18) participating in endurance-based activities, including: distance running, rowing, cycling, swimming, kayaking, and triathlon. The study had to examine at least one source of dietary nitrate, to compare it to no exposure, and to report on any one of the outcomes of interest: power output, time to exhaustion, rating of perceived exertion, time trial performance, distance travelled, work done, VO_2_, VO_2max_, or blood lactate.

The outcome of time to exhaustion is one of significant complexity. Studies varied in use of constant exercise versus incremental/gradual exercise test; this information was not readily available in all studies. As such, whenever possible, we recorded time to exhaustion for controls and nitrate supplementation for available increments and meta-analysed them together. This allowed a comparison (with limitations) of no nitrate to nitrate on time to exhaustion at all potential power outputs. Unfortunately, not enough studies explicity stated incremental versus constant to conduct a sensitivity analysis.

### Data collection and management

We performed data extraction independently and in duplicate using pre-piloted extraction forms. If there was a discrepancy, a third party reviewed the data.

### Risk of bias assessment for RCTs

We judged risk of bias as “low, “high” or “unclear” using the Cochrane Collaboration Risk of Bias Tool [16]. Two independent reviewers evaluated each trial for six aspects: random sequence generation, allocation concealment, blinding of participants and personnel, blinding of outcome assessment, selective reporting and other sources of bias. If all aspects were considered to have “low” risk of bias, we considered the study at “low” risk. If even one aspect or more was considered to have “unclear” risk of bias, we considered the paper at “unclear” risk. Studies with at least one aspect considered to have “high” risk of bias were considered at “high” risk.

### Summary measures of treatment effect and unit of analysis

We analysed data using RevMan 5.3. Expecting heterogeneity among studies, we used a random effects model to pool results and summarize evidence. We evaluated the clinical, methodological and statistical heterogeneity of the included studies to assess whether pooling data was appropriate. We pooled studies using the DerSimonian and Laird method and planned to analyse RCTs and observational studies separately. We presented point estimates as mean difference (MD), with 95% confidence interval (CI).

### Summary measures of treatment effect and unit of analysis

We performed analyses using Review Manager 5.3 (RevMan 5.3). We expected heterogeneity among studies and applied a random effects model to pool relevant results and review the evidence. We presented all outcomes as mean differences (MD) with 95% confidence intervals (CI).

### Assessment of heterogeneity

We used the chi-squared test for homogeneity and the I^2^ statistic to analyse for heterogeneity. We performed subgroups analyses (described later) to explain any heterogeneity observed.

### Publication bias

We inspected the funnel plots for potential publication bias.

### Subgroups

We prespecified the following possible subgroups to explain possible heterogeneity within data:

1. Dose: less than ~ 4 mmol per day versus more than ~ 4 mmol per day;
2. Duration of supplementation: single day versus multiple days;
3. Athletic level: sedentary, recreational athletes, and national−/international-level or elite athletes;
4. Source of dietary nitrate: beetroot juice, nitrate tablet/capsule/beetroot crystals, non-beet foods (pomegranate extract, watercress, red radish) and other (dissolved betaine, high nitrate diet, nitrate-rich gels, sodium nitrate dissolved in water);
5. Age profile: under 20 years of age, 20–29 years of age, and older than 30 years of age;
6. Study design: Randomized or non-randomized comparative study design.

In addition, we evaluated the following subgroups post-hoc:

1. Co-supplementation with one of the following substances: L-arginine, sodium phosphate, caffeine, ultraviolet light A, sodium bicarbonate, N-acetyl-cysteine (NAC);
2. Risk of bias: high and unclear versus low risk of bias.

### Assessment of confidence in pooled effect estimates

We used the Grading of Recommendations Assessment, Development and Evaluation (GRADE) approach to evaluate confidence in effect estimate s[17]. In the GRADE framework, RCTs are considered high-quality evidence, but they can be rated down for risk of bias, imprecision, inconsistency, indirectness or publication bias.

## Results

**Fig. 2:**
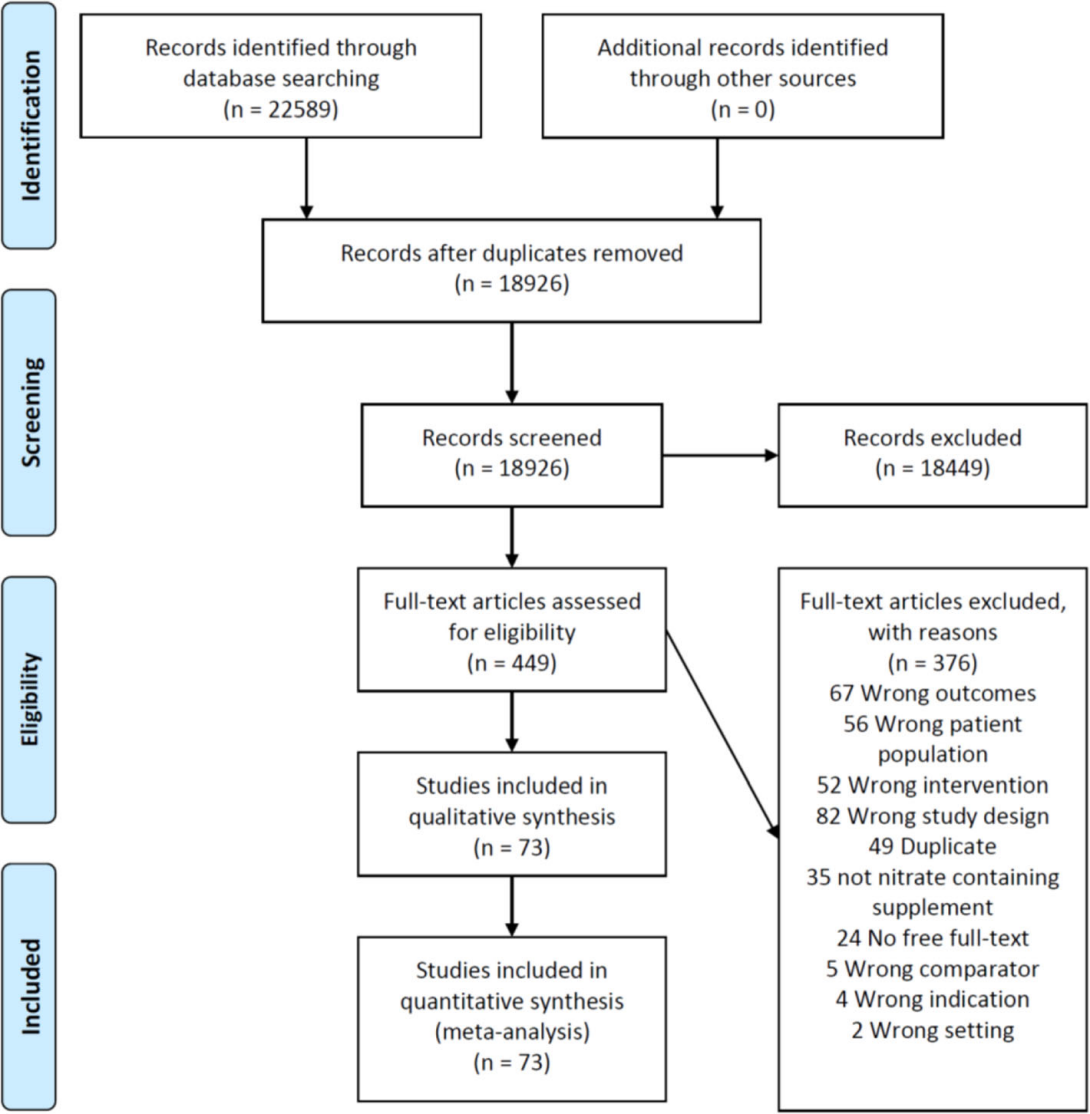
PRISMA flow diagram for study selection

Figure 2 summarizes the screening and study selection process. We identified 17,048 citations for title and abstract screening and we reviewed the full-text of 449 studies. Seventy-three RCTs (*n* = 1061) met inclusion criteria, which we pooled [references provided in Supplemental Table 2]. Supplemental Table 2 summarizes the characteristics of the included studies. These trials were all placebo-controlled, single-centre, and examined nitrate-containing substances in a total of 1061 participants undergoing various endurance-based exercise tests. The participants ranged from sedentary to elite athletes in their level of athletic involvement, with the majority of participants characterized as recreationally active, healthy adults. The trials reported power output (28 studies), time to exhaustion (20 studies), rating of perceived exertion (20 studies), time trial performance (28 studies), distance travelled (2 studies), work done (4 studies), VO_2_ (42 studies), VO_2max_ (10 studies), and blood lactate (23 studies).

Three trials were at low risk of bias, while the remaining trials were at high risk of bias due to unclear descriptions of random sequence generation and/or allocation concealment. Table 1 presents the quality of evidence assessment for each outcome.

**Table 1:**
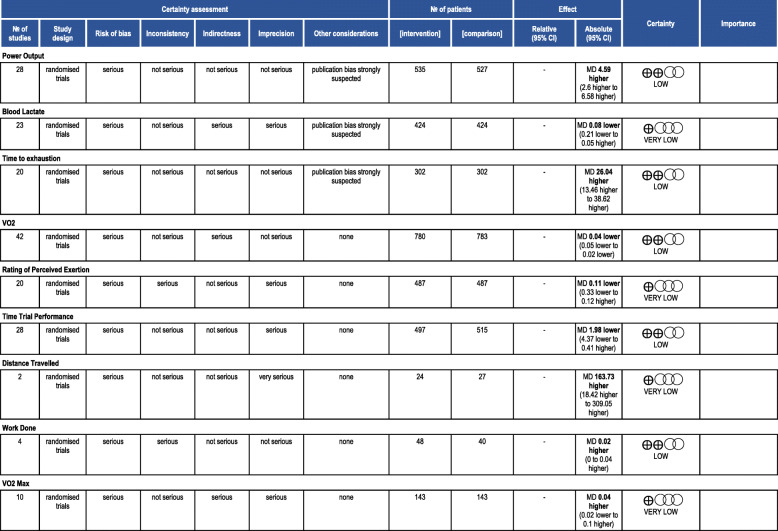
Quality of Evidence - GRADE Assessment

*CI* Confidence interval, *MD* Mean difference

### Power output (Fig. 3)

Nitrates led to an increase in power output compared placebo (MD 4.59 watts, 95% CI [2.6, 6.58], 95%CI, *P* < 0.0001, I^2^ = 0%, low-quality evidence). We downgraded the quality of evidence for serious risk of bias and suspected publication bias (Table 1).

**Fig. 3:**
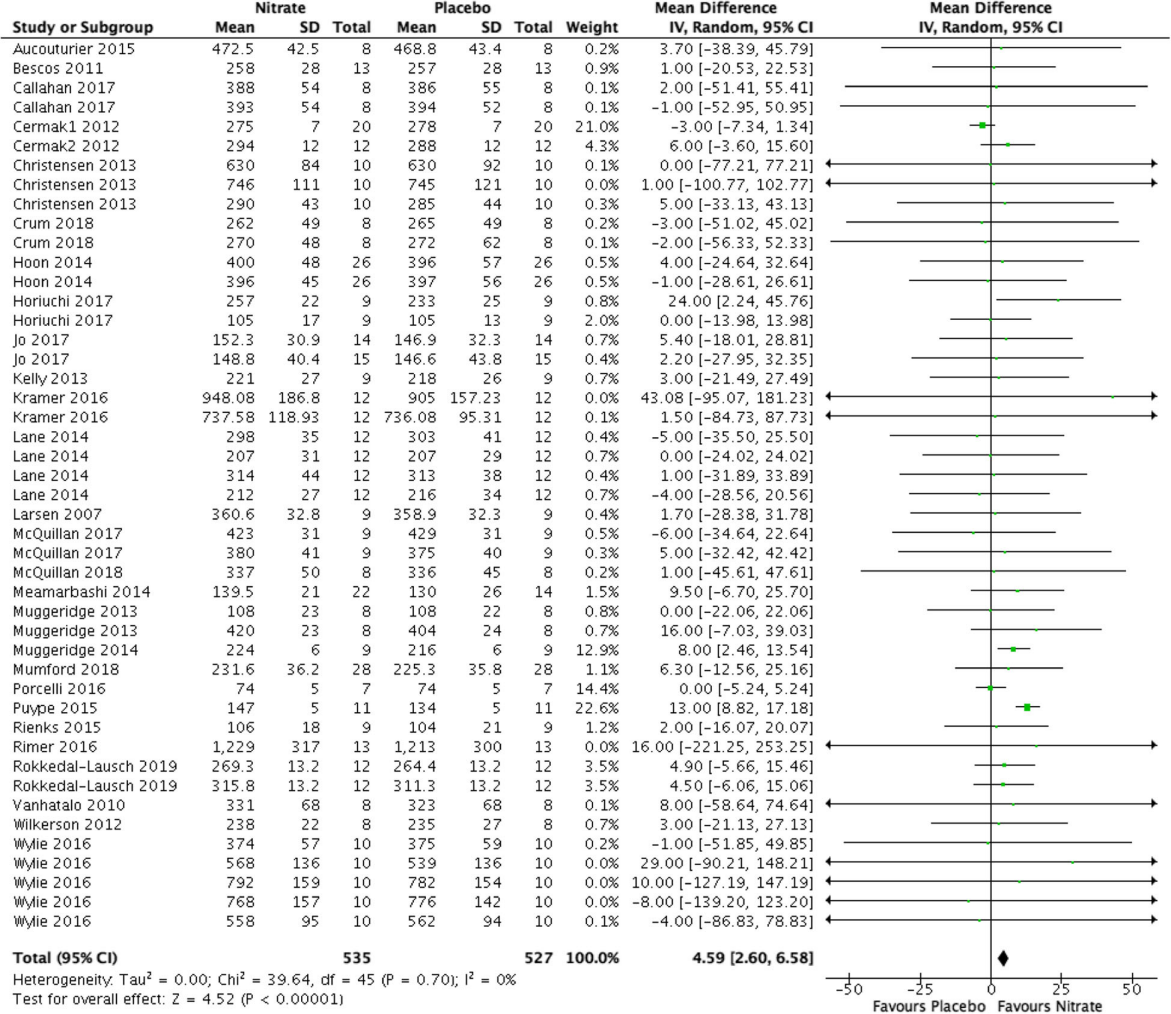
Forest plot for power output in watts for nitrate supplementation versus placebo. Square markers represent mean difference for individual studies, with square size proportional to the weight given to each study in the meta-analysis. Horizontal lines indicate 95% confidence intervals (CI). The solid diamond represents the estimated 95% confidence interval for effect size of all meta-analyzed data

### Time to exhaustion (Fig. 4)

Nitrates increased the time to exhaustion compared to placebo (MD 25.27 s, 95% CI [12.69, 37.84], *P* < 0.00001, I^2^ = 38%, low-quality evidence). We downgraded the quality of evidence for serious risk of bias and suspected publication bias (Table 1).

**Fig. 4:**
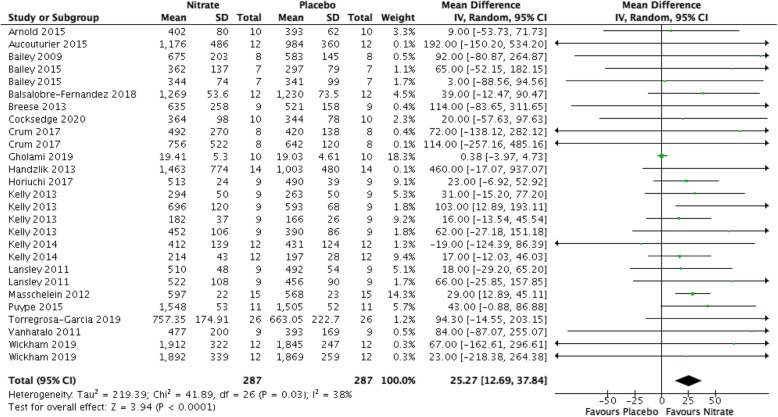
Forest plot for time to exhaustion in seconds for nitrate supplementation versus placebo. Square markers represent mean difference for individual studies, with square size proportional to the weight given to each study in the meta-analysis. Horizontal lines indicate 95% confidence intervals (CI). The solid diamond represents the estimated 95% confidence interval for effect size of all meta-analyzed data

### Distance travelled (Fig. 5)

Participants in the nitrate group had a travelled 163.7 m further compared to participants in the placebo group (MD 163.73 m, 95% CI [18.4, 309.1], *P* = 0.03, I^2^ = 0%, very low-quality evidence). We downgraded the quality of evidence for serious risk of bias and very serious imprecision (Table 1).

**Fig. 5:**
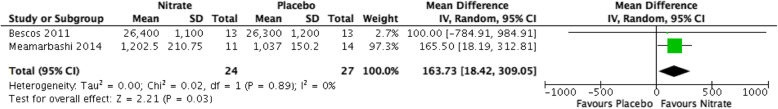
Forest plot for distance travelled in metres for nitrate supplementation versus placebo. Square markers represent mean difference for individual studies, with square size proportional to the weight given to each study in the meta-analysis. Horizontal lines indicate 95% confidence intervals (CI). The solid diamond represents the estimated 95% confidence interval for effect size of all meta-analyzed data

### VO_2_ (Fig. 6)

Participants in the nitrate group had a significant decrease in VO_2_ compared to participants in the placebo group (MD − 0.04 L/min, 95% CI [− 0.05, − 0.02], *P* < 0.0001, I^2^ = 0%, low-quality evidence). We downgraded the quality of evidence for serious risk of bias and serious indirectness (Table 1).

**Fig. 6:**
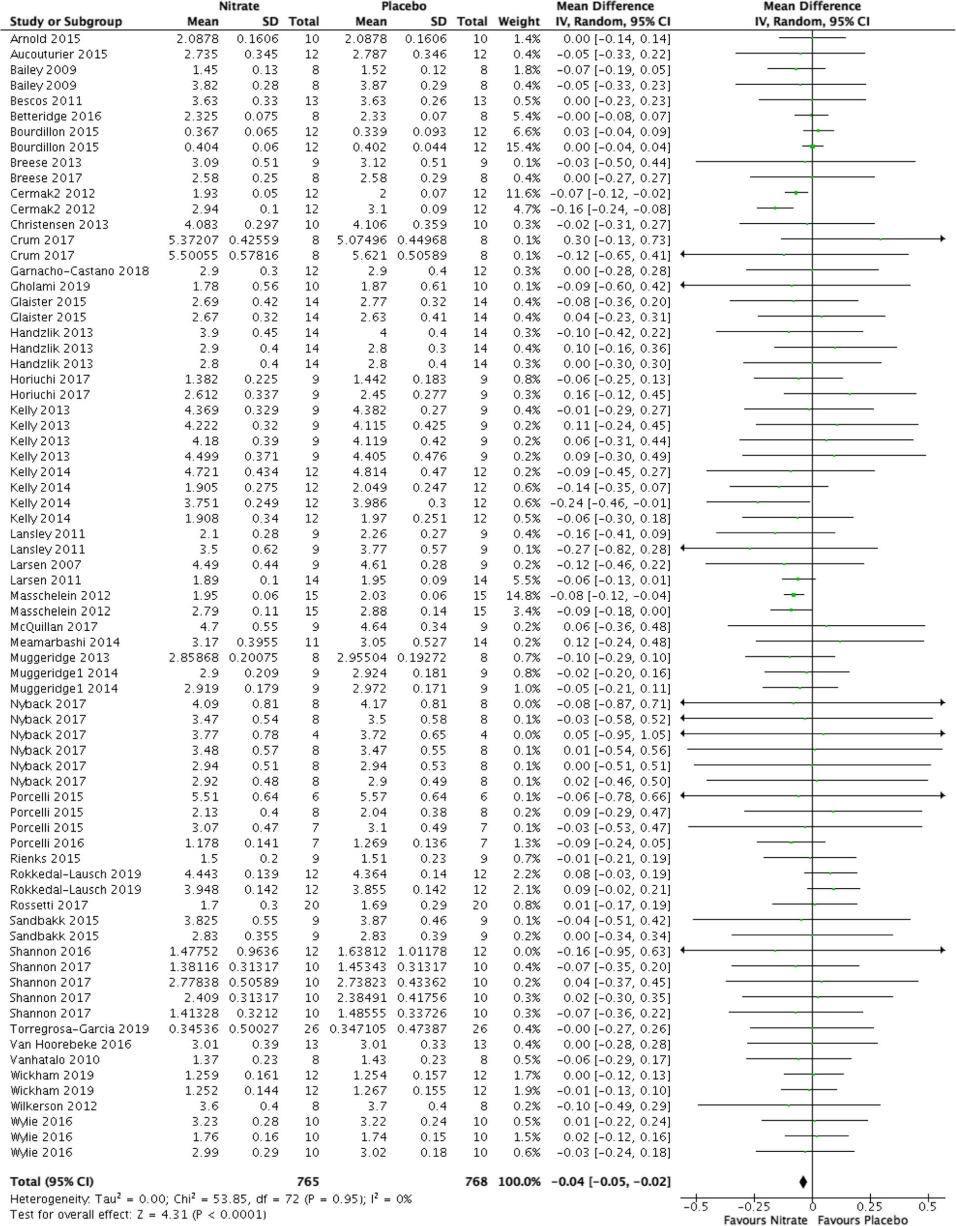
Forest plot for VO_2_ in litres/minute for nitrate supplementation versus placebo. Square markers represent mean difference for individual studies, with square size proportional to the weight given to each study in the meta-analysis. Horizontal lines indicate 95% confidence intervals (CI). The solid diamond represents the estimated 95% confidence interval for effect size of all meta-analyzed data

### VO_2max_ (Fig. 7)

Nitrates did not increase *VO*_*2max*_ compared with placebo (MD 0.04 L/min, 95% CI [− 0.02, 0.10], *P* = 0.23, I^2^ = 0%, very low-quality evidence). We downgraded the quality of evidence for serious risk of bias, serious indirectness, and serious imprecision (Table 1).

**Fig. 7:**
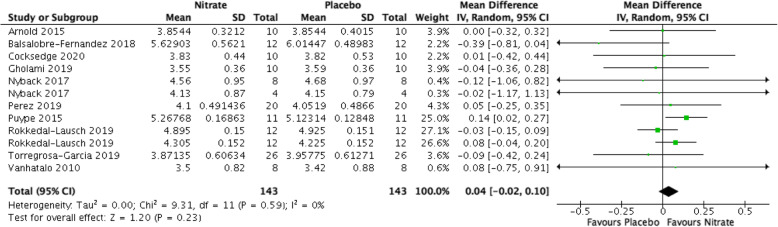
Forest plot for VO_2max_ in litres/minute in kilojoules for nitrate supplementation versus placebo. Square markers represent mean difference for individual studies, with square size proportional to the weight given to each study in the meta-analysis. Horizontal lines indicate 95% confidence intervals (CI). The solid diamond represents the estimated 95% confidence interval for effect size of all meta-analyzed data

### Rating of perceived exertion (Supplemental Fig. 1)

Nitrates did not affect the rating of perceived exertion (Borg scale) (MD -0.11, 95% CI [− 0.34, 0.12], *P* = 0.36, I^2^ = 62%, very low-quality evidence). We downgraded the quality of evidence for serious risk of bias, serious inconsistency, and serious imprecision (Table 1).

### Time trial performance (Supplemental Fig. 2)

Time trials appeared unchanged with and without nitrates (MD − 1.98 s, 95% CI [− 4.37, 0.41], *P* = 0.1, I^2^ = 8%, low-quality evidence). We downgraded the quality of evidence for serious risk of bias and serious imprecision (Table 1).

### Work done (Supplemental Fig. 3)

Nitrates did not significantly increase work done compared with placebo (MD 0.02 kJ, 95% CI [0.0, 0.03], *p* = 0.09, I^2^ = 0%, low-quality evidence). We downgraded the quality of evidence for serious risk of bias and serious inconsistency (Table 1).

### Blood lactate (Supplemental Fig. 4)

Nitrates did not significantly decrease blood lactate compared with placebo (MD − 0.08 mM, 95%CI [− 0.21, 0.05], *P* = 0.22, I^2^ = 12%, very low-quality evidence). We downgraded the quality of evidence for serious risk of bias, serious indirectness, serious imprecision, and suspected publication bias (Table 1).

### Subgroup analyses

We attempted to perform subgroup analyses according to the length of dosing, athletic level, source of dietary nitrate, mean age profile, co-supplementation, and risk of bias. The available data were insufficient to perform subgroup analyses based on the daily dose of nitrate. We identified only one statistically interaction – an interaction (*p* = 0.005) between athletic level and treatment on VO_2_, with no significant effect of nitrate supplementation in elite (MD 0.01, 95% CI [− 0.02, 0.04]) or sedentary athletes (MD 0.06, 95% CI [− 0.16, 0.29]), but a significant effect in recreational athletes (MD -0.05, 95% CI [− 0.07, − 0.03]) (Supplemental Figure 5).

## Discussion

We meta-analyzed the results of 73 trials including participants undergoing various endurance-based exercise tests either with or without nitrate supplementation. Nitrate supplementation improved power output, time to exhaustion, and distance travelled, but did not impact perceived exertion, time trial performance, work done generation of lactate, or VO_2max_. Nitrates led to a reduction in VO2 at different exercise intensities. Therefore, the existing randomized data suggests that nitrate supplementation improves endurance exercise performance by reducing the oxygen cost of the exercise.

Dietary nitrate supplementation may enhance muscle function and exercise performance through the nitrate-nitrite-NO pathway. Dietary inorganic nitrate intake increases circulating nitrate levels, which is then reduced to bioactive nitrite by facultative anaerobic bacteria in the saliva, and subsequently converted into NO in the acidic environment of the stomach. The NO generated via this pathway supplements endogenous NO, produced by oxidation of circulating L-arginine. Further, after nitrate ingestion, plasma nitrate and nitrite concentrations peak after a few hours, and both gradually fall to baseline values in approximately 24 h. Subsequently, many enzymes and proteins, such as deoxyhemoglobin catalyze nitrite to NO in blood and other tissues. This process is facilitated in conditions of low oxygen availability (ischemia and hypoxia), enabling NO production where it is most required; these conditions may exist in skeletal muscle during endurance exercis e[18]. By mediating smooth muscle relaxation, NO promotes vasodilation, increasing oxygen delivery to skeletal muscles [19]. The subsequent improvements in type II muscle fiber function and efficiency have been implicated in dietary nitrate’s positive effects on cardiorespiratory endurance [20].

This postulated mechanism for nitrates to improve muscle contraction efficiency is supported by our finding (albeit with low certainty) that dietary nitrate reduced VO_2_, or the oxygen cost of physical activity. These findings are also congruent with studies suggesting that dietary nitrate supplementation (pure sodium nitrate or beetroot juice) in young, healthy volunteers reduces the submaximal oxygen cost of a given intensity of muscle contractions [21]. Evidence also supports that nitrate supplementation may improve mitochondrial efficiency [21], calcium handling and contractile function [22], translating into higher fraction of oxygenated hemoglobin in muscle, as well as lower rate of whole-body oxygen uptake (VO_2_) in endurance exercise. Our findings indicate that nitrate supplementation’s effect is independent of the maximal oxygen uptake (VO2max), which is regarded as one of the best indicators of an athlete’s physical capacity to work at a higher intensity for a longer period of time, among other factors [23]. As mentioned previously, assessing time to exhaustion is a nuanced outcome. While some studies may test the difference in time over a set distance traveled, others may test distance traveled over increments of time (as seen in graded or incremental exercise testing); both would produce significant differences in absolute values. For instance, an improvement of 25 s might be beneficial over 10 km, but may be even more important at 5 km. Even so, our approach to meta-analysing this outcome at all reported power outputs, along with lack of heterogeneity (I^2^ = 0%), does demonstrate that the increase in time to exhaustion with nitrates is of note.

The significant interaction between athletic level and nitrate treatment may be a spurious finding or may relate to the benefit of nitrates being less dependent on muscle fibre efficiency in elite athletes which may already be optimized. In particular, we cannot draw conclusive inferences from this potential interaction as subgroup analyses are prone to type II errors, especially in a meta-analysis where subgroup analyses are not adjusted for multiplicity.

### Strengths and limitations

Our systematic review and meta-analysis has several strengths: a comprehensive search strategy, the inclusion of randomized data, and a rigorous evaluation of the quality of evidence. However, it also has limitations. First, many studies lacked sufficient methodological details, which led us to adjudicate them at unclear or high risk of bias for evaluated outcomes. Second, the methods of included studies varied in terms of type of exercise test (wide variability in performance variables – in some cases time to exhaustion was a constant load, while in others, it was graded), participants’ athletic background, forms and quantities of nitrate supplementation (wide variability of doses, dose routines and sources used), and co-interventions such as caffeine and ultraviolet-A light. Third, this included trials had small, select study populations and well-monitored adherence, which may limit external validity. Lastly, while we discuss dietary nitrates, with vegetables comprising 80% of naturally available sources, the included trials used a variety of commercially available nitrate supplementary products. These products contain a blend of nitrate-rich foods, extracts and other ingredients, which may modulate nitrate bioavailabilit y[24]. As such, our results should be interpreted in context of commercial nitrate supplementation, rather than ingestion of natural foods.

### Athletes experience and perspective (Reid Coolsaet, Olympic Marathon 2012, 2016)

In 2011 I used beetroot juice in training and in five competitions. My protocol was to drink at least 500 ml of beetroot juice approximately 2–3 h before a competition or run. The competitions all went well as I met or exceeded expectations. Of course, it’s impossible to credit beetroot supplementation alone as there are many variables that lead to successful competitions. I did experience GI distress, which was not problematic in the 10 km distance, slightly problematic competing in the half marathon distance, and problematic over the marathon distance. It was the GI distress that led me to stop supplementing with beetroot juice.

World Athletics lists 5 supplements that improve performance; caffeine, nitrate, creatine, B-alanine and bicarbonate [4]. For endurance sports, the two believed to be most effective are caffeine and nitrate. This meta-analysis suggests that the improvement in endurance activities from nitrates is significant, supporting the endorsement by the World Athletics Organization.

With the knowledge that multiple-day ingestion is effective and high nitrate doses are available commercially in smaller volumes, as an elite athlete I am interested in testing nitrate supplementation again. Future research should establish the best dosing strategy including how long before competition one can stop supplementing without losing the benefit.

## Conclusions

Based on very low- to moderate-quality, RCT data, this systematic review and meta-analysis suggests that dietary nitrate supplementation improves performance during endurance sports. This is especially evident when evaluating important outcomes, such as power output, time to exhaustion and distance traveled. However, given its mixed effects on explanatory variables, like blood lactate and VO_2_, further research is needed to determine the specific means by which nitrate supplementation impacts physical endurance and establish the optimal dosing strategy accounting for adverse GI effects that may accompany some formulations.

## Supplementary Information

**Additional file 1: Supplemental Figure 1.** Forest plot for rating of perceived exertion for nitrate supplementation versus placebo. Square markers represent mean difference for individual studies, with square size proportional to the weight given to each study in the meta-analysis. Horizontal lines indicate 95% confidence intervals (CI). The solid diamond represents the estimated 95% confidence interval for effect size of all meta-analyzed data. **Supplemental Figure 2.** Forest plot for time trial performance of nitrate supplementation versus placebo. Square markers represent mean difference for individual studies, with square size proportional to the weight given to each study in the meta-analysis. Horizontal lines indicate 95% confidence intervals (CI). The solid diamond represents the estimated 95% confidence interval for effect size of all meta-analyzed data. **Supplemental Figure 3.** Forest plot for work done of nitrate supplementation versus placebo. Square markers represent mean difference for individual studies, with square size proportional to the weight given to each study in the meta-analysis. Horizontal lines indicate 95% confidence intervals (CI). The solid diamond represents the estimated 95% confidence interval for effect size of all meta-analyzed data. **Supplemental Figure 4.** Forest plot of blood lactate levels with nitrate supplementation versus placebo. Square markers represent mean difference for individual studies, with square size proportional to the weight given to each study in the meta-analysis. Horizontal lines indicate 95% confidence intervals (CI). The solid diamond represents the estimated 95% confidence interval for effect size of all meta-analyzed data. **Supplemental Figure 5.** Forest plot with subgroup analysis of VO2 with nitrate supplementation versus placebo, based on athletic level. Square markers represent mean difference for individual studies, with square size proportional to the weight given to each study in the meta-analysis. Horizontal lines indicate 95% confidence intervals (CI). The solid diamond represents the estimated 95% confidence interval for effect size of all meta-analyzed data.

**Additional file 2: Supplementary Table 1.** Search strategies and search terms used with various databases.

**Additional file 3: Supplemental Table 2.** Study Characteristics of Included Randomized Controlled Trials

## Data Availability

The authors will consider written requests for data.
