# Supplementary material for: The effects of dietary nitrate supplementation on endurance exercise performance and cardiorespiratory measures in healthy adults: a systematic review and meta-analysis
Source: J Int Soc Sports Nutr. 2021 Jul 9;18:55. doi: 10.1186/s12970-021-00450-4 (PMC8268374; doi:10.1186/s12970-021-00450-4)
Supplement: Supplementary file 1 — Additional file 1: Supplemental Figure 1. Forest plot for rating of perceived exertion for nitrate supplementation versus placebo. Square markers represent mean difference for individual studies, with square size proportional to the weight given to each study in the meta-analysis. Horizontal lines indicate 95% confidence intervals (CI). The solid diamond represents the estimated 95% confidence interval for effect size of all meta-analyzed data. Supplemental Figure 2. Forest plot for time trial performance of nitrate supplementation versus placebo. Square markers represent mean difference for individual studies, with square size proportional to the weight given to each study in the meta-analysis. Horizontal lines indicate 95% confidence intervals (CI). The solid diamond represents the estimated 95% confidence interval for effect size of all meta-analyzed data. Supplemental Figure 3. Forest plot for work done of nitrate supplementation versus placebo. Square markers represent mean difference for individual studies, with square size proportional to the weight given to each study in the meta-analysis. Horizontal lines indicate 95% confidence intervals (CI). The solid diamond represents the estimated 95% confidence interval for effect size of all meta-analyzed data. Supplemental Figure 4. Forest plot of blood lactate levels with nitrate supplementation versus placebo. Square markers represent mean difference for individual studies, with square size proportional to the weight given to each study in the meta-analysis. Horizontal lines indicate 95% confidence intervals (CI). The solid diamond represents the estimated 95% confidence interval for effect size of all meta-analyzed data. Supplemental Figure 5. Forest plot with subgroup analysis of VO2 with nitrate supplementation versus placebo, based on athletic level. Square markers represent mean difference for individual studies, with square size proportional to the weight given to each study in the meta-analysis. Horizontal [file 12970_2021_450_MOESM1_ESM.docx]

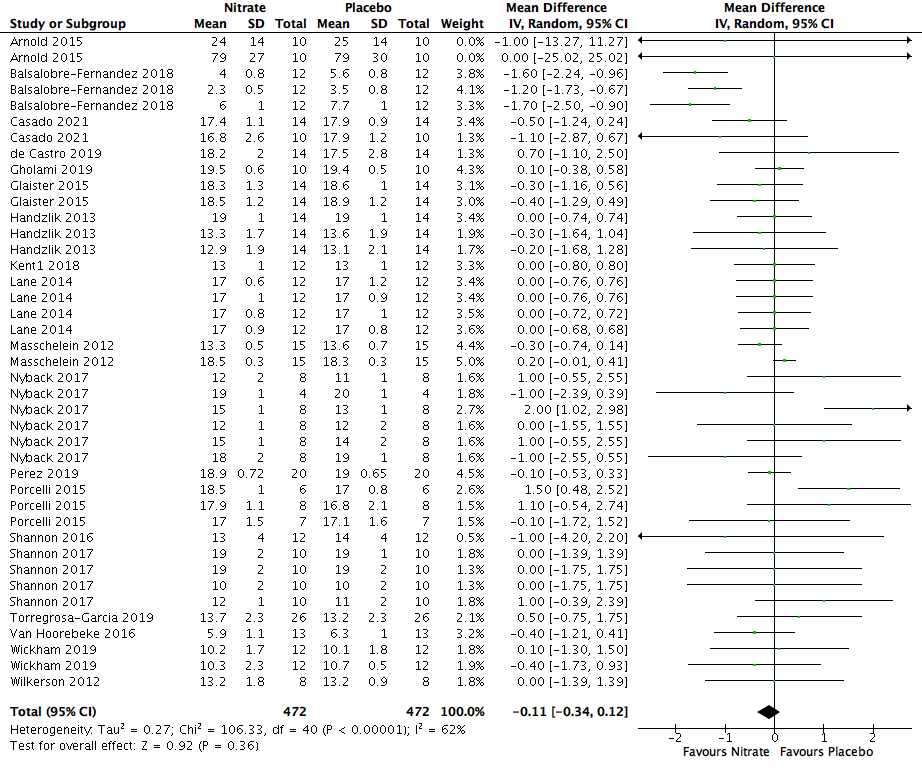


Supplemental Figure 1. Forest plot for rating of perceived exertion for nitrate supplementation versus placebo. Square markers represent mean difference for individual studies, with square size proportional to the weight given to each study in the meta-analysis. Horizontal lines indicate 95% confidence intervals (CI). The solid diamond represents the estimated 95% confidence interval for effect size of all meta-analyzed data.


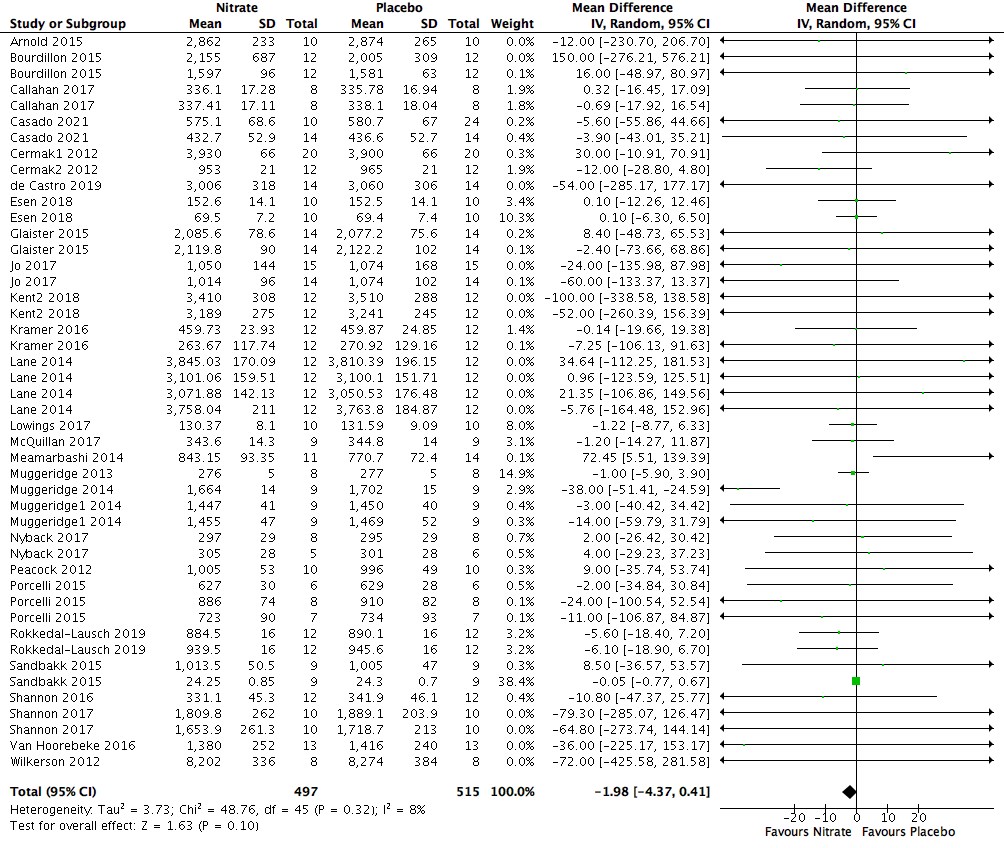
Supplemental Figure 2. Forest plot for time trial performance of nitrate supplementation versus placebo. Square markers represent mean difference for individual studies, with square size proportional to the weight given to each study in the meta-analysis. Horizontal lines indicate 95% confidence intervals (CI). The solid diamond represents the estimated 95% confidence interval for effect size of all meta-analyzed data.

**
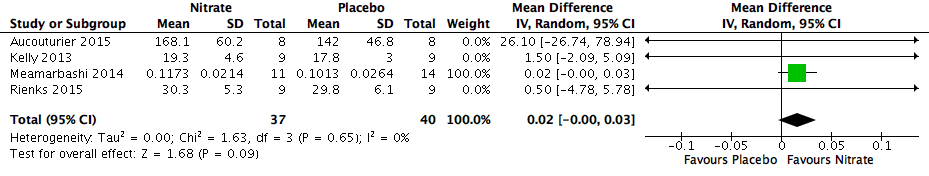
**

Supplemental Figure 3. Forest plot for work done of nitrate supplementation versus placebo. Square markers represent mean difference for individual studies, with square size proportional to the weight given to each study in the meta-analysis. Horizontal lines indicate 95% confidence intervals (CI). The solid diamond represents the estimated 95% confidence interval for effect size of all meta-analyzed data.


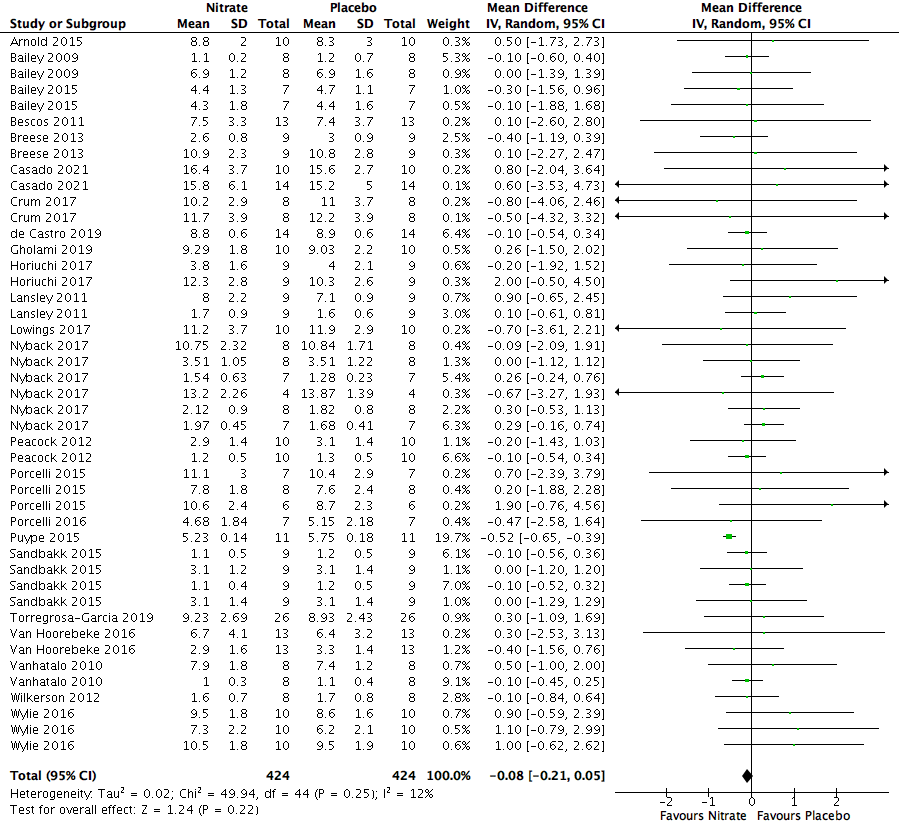


Supplemental Figure 4. Forest plot of blood lactate levels with nitrate supplementation versus placebo. Square markers represent mean difference for individual studies, with square size proportional to the weight given to each study in the meta-analysis. Horizontal lines indicate 95% confidence intervals (CI). The solid diamond represents the estimated 95% confidence interval for effect size of all meta-analyzed data.

**
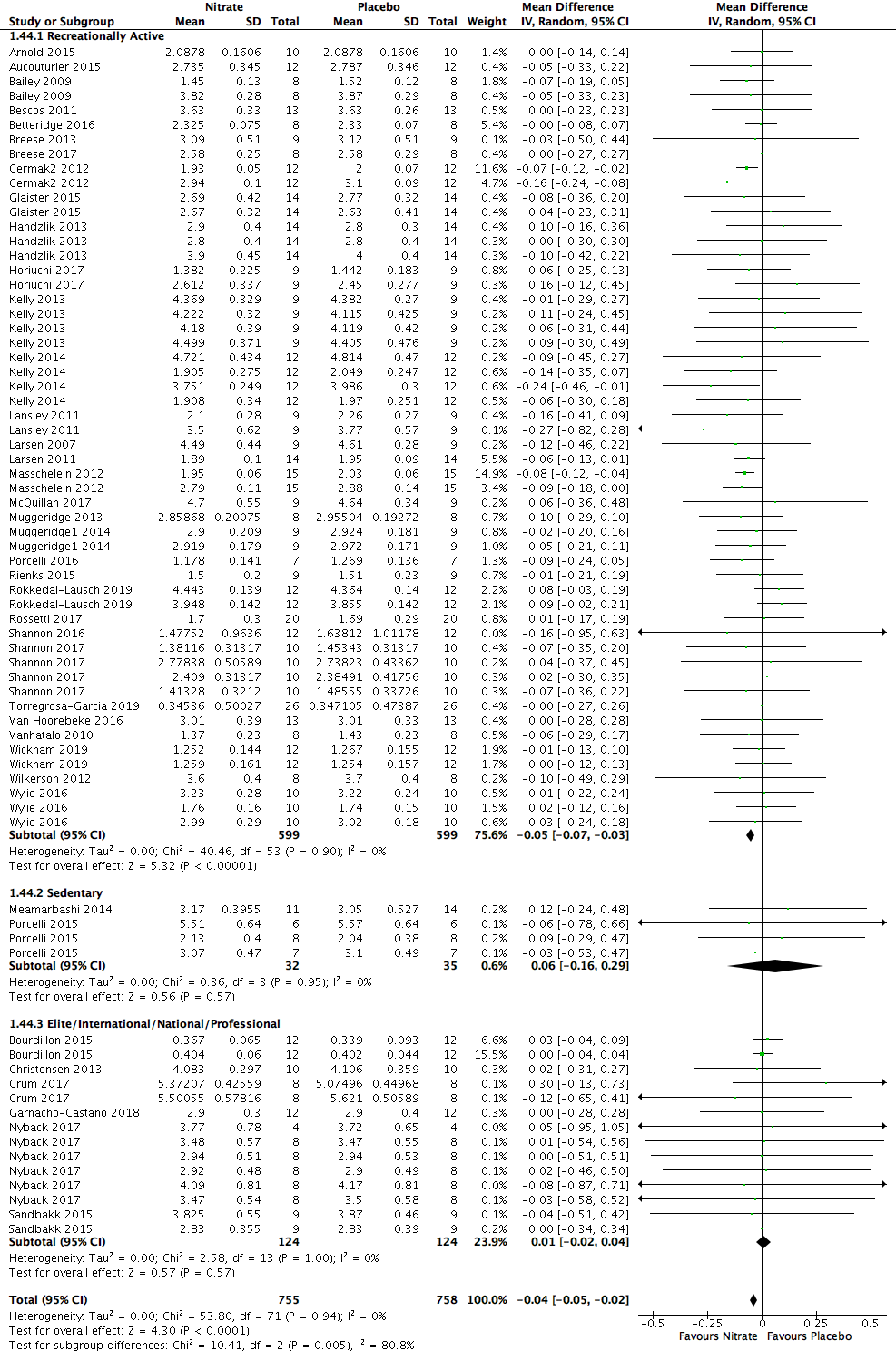
**Supplemental Figure 5. Forest plot with subgroup analysis of VO2 with nitrate supplementation versus placebo, based on athletic level. Square markers represent mean difference for individual studies, with square size proportional to the weight given to each study in the meta-analysis. Horizontal lines indicate 95% confidence intervals (CI). The solid diamond represents the estimated 95% confidence interval for effect size of all meta-analyzed data.
