# Supplementary material for: The effects of dietary nitrate supplementation on endurance exercise performance and cardiorespiratory measures in healthy adults: a systematic review and meta-analysis
Source: J Int Soc Sports Nutr. 2021 Jul 9;18:55. doi: 10.1186/s12970-021-00450-4 (PMC8268374; doi:10.1186/s12970-021-00450-4)

Supplementary Table 1: Search strategies and search terms used with various databases.

**OVID Medline Epub Ahead of Print, In-Process & Other Non-Indexed Citations, Ovid MEDLINE(R) Daily and Ovid MEDLINE(R) 1946 to Present**

| 1 | nitrate*.mp. [mp=title, abstract, original title, name of substance word, subject heading word, floating sub-heading word, keyword heading word, organism supplementary concept word, protocol supplementary concept word, rare disease supplementary concept word, unique identifier, synonyms] | 80502 |
| --- | --- | --- |
| 2 | exp NITRATES/ | 34611 |
| 3 | exp Beta vulgaris/ | 2174 |
| 4 | exp "Fruit and Vegetable Juices"/ | 2186 |
| 5 | beta vulgaris.mp. [mp=title, abstract, original title, name of substance word, subject heading word, floating sub-heading word, keyword heading word, organism supplementary concept word, protocol supplementary concept word, rare disease supplementary concept word, unique identifier, synonyms] | 3142 |
| 6 | juice*.mp. [mp=title, abstract, original title, name of substance word, subject heading word, floating sub-heading word, keyword heading word, organism supplementary concept word, protocol supplementary concept word, rare disease supplementary concept word, unique identifier, synonyms] | 50221 |
| 7 | exp Plant Roots/ | 65855 |
| 8 | plant root*.mp. [mp=title, abstract, original title, name of substance word, subject heading word, floating sub-heading word, keyword heading word, organism supplementary concept word, protocol supplementary concept word, rare disease supplementary concept word, unique identifier, synonyms] | 55625 |
| 9 | beet*.mp. [mp=title, abstract, original title, name of substance word, subject heading word, floating sub-heading word, keyword heading word, organism supplementary concept word, protocol supplementary concept word, rare disease supplementary concept word, unique identifier, synonyms] | 21802 |
| 10 | or/1-9 | 218684 |
| 11 | exp Physical Endurance/ | 34564 |
| 12 | physical endurance.mp. [mp=title, abstract, original title, name of substance word, subject heading word, floating sub-heading word, keyword heading word, organism supplementary concept word, protocol supplementary concept word, rare disease supplementary concept word, unique identifier, synonyms] | 19912 |
| 13 | Physical Fitness/ or Oxygen Consumption/ or Exercise Test/ or VO2 max.mp. or Exercise/ or Physical Exertion/ | 314204 |
| 14 | physical fitness.mp. [mp=title, abstract, original title, name of substance word, subject heading word, floating sub-heading word, keyword heading word, organism supplementary concept word, protocol supplementary concept word, rare disease supplementary concept word, unique identifier, synonyms] | 32863 |
| 15 | oxygen consumption.mp. [mp=title, abstract, original title, name of substance word, subject heading word, floating sub-heading word, keyword heading word, organism supplementary concept word, protocol supplementary concept word, rare disease supplementary concept word, unique identifier, synonyms] | 120836 |
| 16 | exercise test.mp. [mp=title, abstract, original title, name of substance word, subject heading word, floating sub-heading word, keyword heading word, organism supplementary concept word, protocol supplementary concept word, rare disease supplementary concept word, unique identifier, synonyms] | 70258 |
| 17 | exercise*.mp. [mp=title, abstract, original title, name of substance word, subject heading word, floating sub-heading word, keyword heading word, organism supplementary concept word, protocol supplementary concept word, rare disease supplementary concept word, unique identifier, synonyms] | 401534 |
| 18 | physical exertion.mp. [mp=title, abstract, original title, name of substance word, subject heading word, floating sub-heading word, keyword heading word, organism supplementary concept word, protocol supplementary concept word, rare disease supplementary concept word, unique identifier, synonyms] | 58058 |
| 19 | endurance*.mp. [mp=title, abstract, original title, name of substance word, subject heading word, floating sub-heading word, keyword heading word, organism supplementary concept word, protocol supplementary concept word, rare disease supplementary concept word, unique identifier, synonyms] | 40927 |
| 20 | endurance activit*.mp. [mp=title, abstract, original title, name of substance word, subject heading word, floating sub-heading word, keyword heading word, organism supplementary concept word, protocol supplementary concept word, rare disease supplementary concept word, unique identifier, synonyms] | 185 |
| 21 | athlet*.mp. [mp=title, abstract, original title, name of substance word, subject heading word, floating sub-heading word, keyword heading word, organism supplementary concept word, protocol supplementary concept word, rare disease supplementary concept word, unique identifier, synonyms] | 93760 |
| 22 | exp Athletes/ | 14550 |
| 23 | or/11-22 | 602395 |
| 24 | 10 and 23 | 3661 |
| 25 | animals/ not humans/ | 4783755 |
| 26 | 24 not 25 | 3086 |
| 27 | limit 26 to (case reports or letter) | 91 |
| 28 | 26 not 27 | 2995 |
| 29 | limit 28 to yr="2019 -Current" | 309 |

**Embase <1974 to 2021 April 26>**

| 1 | exp endurance training/ or exp endurance/ or exp endurance sport/ | 32969 |
| --- | --- | --- |
| 2 | endurance*.mp. | 50714 |
| 3 | (endurance training or endurance sport).mp. [mp=title, abstract, heading word, drug trade name, original title, device manufacturer, drug manufacturer, device trade name, keyword, floating subheading word, candidate term word] | 12586 |
| 4 | endurance activit*.mp. [mp=title, abstract, heading word, drug trade name, original title, device manufacturer, drug manufacturer, device trade name, keyword, floating subheading word, candidate term word] | 264 |
| 5 | exp fitness/ or exp cardiorespiratory fitness/ | 43604 |
| 6 | fitness.mp. [mp=title, abstract, heading word, drug trade name, original title, device manufacturer, drug manufacturer, device trade name, keyword, floating subheading word, candidate term word] | 106602 |
| 7 | cardiorespiratory fitness.mp. [mp=title, abstract, heading word, drug trade name, original title, device manufacturer, drug manufacturer, device trade name, keyword, floating subheading word, candidate term word] | 9331 |
| 8 | (exercise or oxygen consumption or aerobic capacity or exercise test).mp. [mp=title, abstract, heading word, drug trade name, original title, device manufacturer, drug manufacturer, device trade name, keyword, floating subheading word, candidate term word] | 612365 |
| 9 | exp exercise/ or exp oxygen consumption/ or exp aerobic capacity/ or exp exercise test/ or VO2 max.mp. or exp running/ or exp oxygen/ | 1077685 |
| 10 | exp athlete/ | 63338 |
| 11 | athlet*.mp. [mp=title, abstract, heading word, drug trade name, original title, device manufacturer, drug manufacturer, device trade name, keyword, floating subheading word, candidate term word] | 100251 |
| 12 | or/1-11 | 1336371 |
| 13 | exp beet/ or beet*.mp. | 26230 |
| 14 | exp nitrate/ | 56253 |
| 15 | nitrate*.mp. | 116084 |
| 16 | exp beetroot juice/ | 331 |
| 17 | beetroot juice.mp. [mp=title, abstract, heading word, drug trade name, original title, device manufacturer, drug manufacturer, device trade name, keyword, floating subheading word, candidate term word] | 586 |
| 18 | juice*.mp. or exp "fruit and vegetable juice"/ or exp vegetable juice/ | 51670 |
| 19 | or/13-18 | 191882 |
| 20 | 12 and 19 | 16489 |
| 21 | case report/ | 2635501 |
| 22 | limit 20 to letter | 128 |
| 23 | 20 and 21 | 330 |
| 24 | 22 or 23 | 435 |
| 25 | 20 not 24 | 16054 |
| 26 | animals/ not humans/ | 984203 |
| 27 | 25 not 26 | 15459 |
| 28 | limit 27 to yr="2019 -Current" | 2025 |

**CENTRAL:**

ID Search Hits

| 1 | MeSH descriptor: [Physical Endurance] explode all trees | 5593 |
| --- | --- | --- |
| 2 | endurance activit* | 2182 |
| 3 | endurance | 8103 |
| 4 | MeSH descriptor: [Oxygen Consumption] explode all trees | 6235 |
| 5 | MeSH descriptor: [Sports Nutritional Physiological Phenomena] explode all trees | 187 |
| 6 | MeSH descriptor: [Cardiorespiratory Fitness] explode all trees | 91 |
| 7 | MeSH descriptor: [Exercise] explode all trees | 21034 |
| 8 | MeSH descriptor: [Nitrates] explode all trees | 893 |
| 9 | MeSH descriptor: [Beta vulgaris] explode all trees | 94 |
| 10 | MeSH descriptor: [Fruit and Vegetable Juices] explode all trees | 152 |
| 11 | beet* | 555 |
| 12 | plant root | 455 |
| 13 | #1 or #2 or #3 or #4 or #5 or #6 or #7 | 30209 |
| 14 | #8 or #9 or #10 or #11 or #12 | 1897 |
| 15 | #13 and #14 | 216 |

**CINAHL:**

| **#** | **Query** | **Limiters/Expanders** | **Last Run Via** | **Results** |
| --- | --- | --- | --- | --- |
| S19 | S15 AND S17 | Search modes - Boolean/Phrase | Interface - EBSCOhost Research Databases  Search Screen - Advanced Search  Database - CINAHL | Display |
| S18 | S15 AND S17 | Search modes - Boolean/Phrase | Interface - EBSCOhost Research Databases  Search Screen - Advanced Search  Database - CINAHL | Display |
| S17 | S11 OR S12 OR S13 OR S14 OR S16 | Search modes - Boolean/Phrase | Interface - EBSCOhost Research Databases  Search Screen - Advanced Search  Database - CINAHL | Display |
| S16 | juice* | Search modes - Boolean/Phrase | Interface - EBSCOhost Research Databases  Search Screen - Advanced Search  Database - CINAHL | Display |
| S15 | S1 OR S2 OR S3 OR S4 OR S5 OR S6 OR S7 OR S8 OR S9 OR S10 | Search modes - Boolean/Phrase | Interface - EBSCOhost Research Databases  Search Screen - Advanced Search  Database - CINAHL | Display |
| S14 | nitrate* | Search modes - Boolean/Phrase | Interface - EBSCOhost Research Databases  Search Screen - Advanced Search  Database - CINAHL | Display |
| S13 | (MH "Nitrates+") | Search modes - Boolean/Phrase | Interface - EBSCOhost Research Databases  Search Screen - Advanced Search  Database - CINAHL | Display |
| S12 | beet* | Search modes - Boolean/Phrase | Interface - EBSCOhost Research Databases  Search Screen - Advanced Search  Database - CINAHL | Display |
| S11 | (MH "Beet") OR (MH "Fruit Juices+") | Search modes - Boolean/Phrase | Interface - EBSCOhost Research Databases  Search Screen - Advanced Search  Database - CINAHL | Display |
| S10 | vo2 max | Search modes - Boolean/Phrase | Interface - EBSCOhost Research Databases  Search Screen - Advanced Search  Database - CINAHL | Display |
| S9 | (MH "Aerobic Capacity") OR (MH "Aerobic Exercises+") | Search modes - Boolean/Phrase | Interface - EBSCOhost Research Databases  Search Screen - Advanced Search  Database - CINAHL | Display |
| S8 | (MH "Oxygen Consumption+") | Search modes - Boolean/Phrase | Interface - EBSCOhost Research Databases  Search Screen - Advanced Search  Database - CINAHL | Display |
| S7 | "athlet*" | Search modes - Boolean/Phrase | Interface - EBSCOhost Research Databases  Search Screen - Advanced Search  Database - CINAHL | Display |
| S6 | (MH "Athletes+") | Search modes - Boolean/Phrase | Interface - EBSCOhost Research Databases  Search Screen - Advanced Search  Database - CINAHL | Display |
| S5 | exercise* | Search modes - Boolean/Phrase | Interface - EBSCOhost Research Databases  Search Screen - Advanced Search  Database - CINAHL | Display |
| S4 | (MH "Physical Activity") OR (MH "Exercise+") OR (MH "Exercise Test") | Search modes - Boolean/Phrase | Interface - EBSCOhost Research Databases  Search Screen - Advanced Search  Database - CINAHL | Display |
| S3 | fitness | Search modes - Boolean/Phrase | Interface - EBSCOhost Research Databases  Search Screen - Advanced Search  Database - CINAHL | Display |
| S2 | (MH "Cardiorespiratory Fitness") OR (MH "Physical Fitness+") OR (MH "Athletic Training Programs") | Search modes - Boolean/Phrase | Interface - EBSCOhost Research Databases  Search Screen - Advanced Search  Database - CINAHL | Display |
| S1 | (MH "Endurance Sports") OR (MH "Physical Endurance+") OR (MH "Triathlon") OR "endurance" | Search modes - Boolean/Phrase | Interface - EBSCOhost Research Databases  Search Screen - Advanced Search  Database - CINAHL | Display |

**Web of Science**
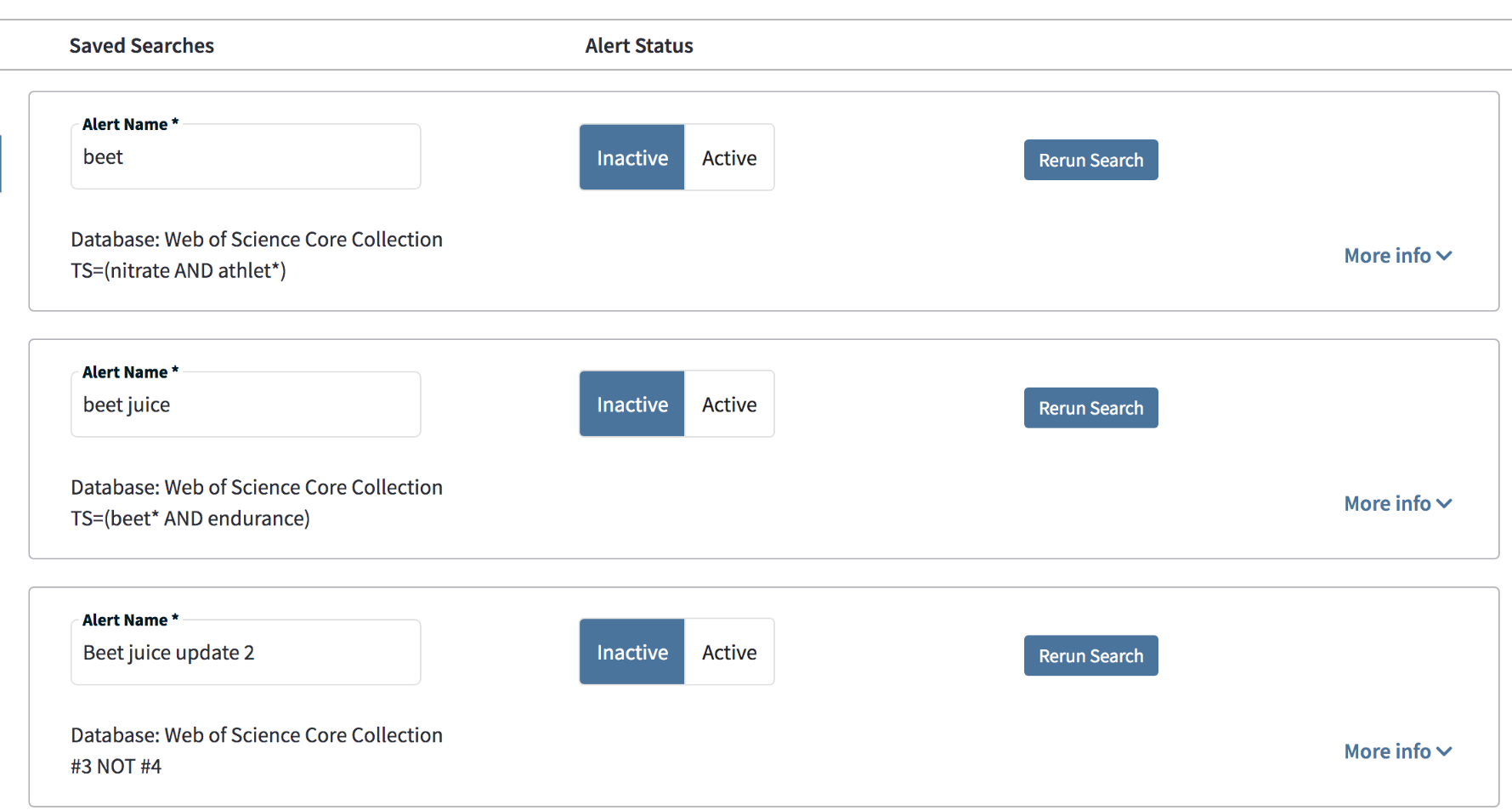

Supplement: Supplementary file 2 — Additional file 2: Supplementary Table 1. Search strategies and search terms used with various databases. [file 12970_2021_450_MOESM2_ESM.docx]
