# Supplementary material for: The effects of dietary nitrate supplementation on endurance exercise performance and cardiorespiratory measures in healthy adults: a systematic review and meta-analysis
Source: J Int Soc Sports Nutr. 2021 Jul 9;18:55. doi: 10.1186/s12970-021-00450-4 (PMC8268374; doi:10.1186/s12970-021-00450-4)
Supplement: Supplementary file 3 — Additional file 3: Supplemental Table 2. Study Characteristics of Included Randomized Controlled Trials [file 12970_2021_450_MOESM3_ESM.docx]

Supplemental Table 2 – Study Characteristics of Included Randomized Controlled Trials

| **Study ID** | **Intervention** | **Control** | **Co-Supplement** | **Inclusion Criteria;  Level of Sport;  Mean Age (Standard Deviation)** | **Outcomes Measured** |
| --- | --- | --- | --- | --- | --- |
| Amano 2018 [1]  (N=8) | Beetroot juice (70ml), twice a day on days 1-2; once on day 3.  2 hours pre-exercise. | Placebo juice (70ml), twice a day on days 1-2; once on day 3.  2 hours pre-exercise. | None. | Healthy adults (18 yrs or older);  Recreationally active;  24 (4) | Body Temperature, Vascular Conductance |
| Arnold 2015 [2]  (N=10) | Beetroot juice (70ml) – once.  2.5 hours pre-exercise. | Placebo juice (70ml) – once.  2.5 hours pre-exercise. | None. | Adults (18 yrs or older);  Well-trained runners from recreational running clubs;  37 (13) | VO2 Max, Time for Exhaustion, Performance for Time Trials, Performance for Graded Exercise Tests, Heart Rate, Lactate Levels, Self-Perceived Exertion |
| Aucouturier 2015 [3]  (N=12) | Beetroot juice (500ml) with an average nitrate content of 680 mg/L. Once a day for 3 days.  2 hours pre-exercise. | Apple/black currant juice (500ml) with an average nitrate content of <5 mg/L. Once a day for 3 days.  2 hours pre-exercise. | None. | Non-smoking adults (18 yrs or older);  Recreational participation in team sports;  22.8 (3.1) | Gas exchange parameters (VCO2, VO2), Hemodynamic parameters (blood flow, blood pressure, vascular conductance and diameter) |
| Bailey 2009 [4]  (N=16) | Beetroot juice (500ml). Once a day for 6 days. | Black currant juice. Once a day for 6 days | None. | Healthy adults (18 yrs or older);  Recreationally active;  26 (7) | Muscle Fractional O2 Extraction, Time for Exhaustion, O2 Uptake Slow Component, Pulmonary O2 Uptake, Blood Nitrate Level, VO2 Max, Performance for Time Trials |
| Bailey 2015 [5]  (N=14) | Beetroot juice (70ml). Twice a day for 3 days. Once a day on test days.  2.5 hours pre-exercise. | NaCl (0.1 mmol/kg body mass). Twice a day for 3 days. Once a day on test days.  2.5 hours pre-exercise. | None. | Healthy adults (18 yrs or older);  Recreationally active;  21 (2) | Oxyhemoglobin concentration, Limit of Tolerance |
| Balsalobre-Fernandez 2018 [6]  (N=12) | Beetroot juice (70mL) for 15 days. | Placebo juice (70mL) for 15 days. | None. | Male adults (18 yrs or older);  Elite middle and long-distance runners;  Nitrate Group: 27.3 (7.8)  Placebo Group: 24.2 (2.9) | Leg Stiffness, Heart Rate, VO2 Max, Rate of Perceived Exertion, Respiratory Exchange Ratio |
| Bescos 2011 [7]  (N=26) | Sodium nitrate (10 mg/kg of body mass) dissolved in 250 mL of water. Once a day for 3 days.  Final dose 3 hours pre-exercise test. | NaCL dissolved in 250 mL of water. Once a day for 3 days.  Final dose 3 hours pre-exercise test. | None. | Male adults (18 yrs or older);  Non-professional cyclists and triathletes;  32.6 (5.6) | VO2 Max, VCO2, Minute Ventilation, RER. HR, Lactate Levels, |
| Betteridge 2015 [8]  (N=16) | Beetroot juice (140ml) – one dose.  2.5 hours pre-exercise test. | Beetroot juice depleted of nitrate (140ml) – one dose.  2.5 hours pre-exercise test. | None. | Male adults (18 yrs or older);  Recreationally active;  27 (1) | VO2, VCO2, Respiratory Exchange Ratio, Muscle glycogen, Muscle lactate, ATP, Phosphocreatine and Creatine levels |
| Bourdillon 2015 [9]  (N=24) | Sodium nitrate capsules ( 0.1mmol/kg/day) for 3 days. | Placebo capsules for 3 days . | None. | Male adults (18 yrs or older); Regional and/or national competition cyclists;  31 (7) | Ventilation, VO2, VCO2, Blood Pressure, Systolic Blood Pressure, Diastolic Blood Pressure, Heart Rate, 15 kilometer time-trial cycling |
| Breese 2013 [10]  (N=18) | Beetroot juice (140ml) once daily for 6 days.  Final dose 2 hours pre-exercise test. | Placebo juice (140ml) once daily for 6 days.  Final dose 2 hours pre-exercise test. | None. | Adults (18 yrs or older); Recreationally active cyclists;  30 (6) | [Hb tot], [HHB], VO2 kinetics, Blood lactate, heart rate, Pulmonary O2 uptake |
| Breese 2017 [11]  (N=16) | Beetroot juice (140 mL) for 4 days. | Nitrate deprived beetroot juice (140ml) for 4 days. | None. | Non-smoking, male adults (18 yrs or older) free of cardiovascular, respiratory, and metabolic disease;  Physically active recreational cyclists;  24 (6) | VO2, Kinetics and amplitude of absolute [Hb + Mb] |
| Callahan 2017 [12]  (N=16) | Beetroot crystal (300 mg nitrate) for 3 days. | Placebo crystals for 3 days. | Bicarbonate gelatin or placebo gelatin capsules. | Non-smoking, adults (18 yrs or older) free of cardiovascular, respiratory, and metabolic disease;  Physically active recreational cyclists;  34 (6) | Plasma Nitrate, Heart Rate, Power Output, Time Trial Performance |
| Cermak 2012 [13]  (N=22) | Beetroot juice (140ml) – one dose.  2.5 hours pre- exercise test. | Placebo juioce (140ml) – one dose.  2.5 hours pre- exercise test. | None. | Male adults (18 yrs or older);  Recreationally trained cyclists or triathletes;  26 (1) | Plasma Nitrate, Heart Rate, Power Output, Time Trial Performance, Plasma Nitrite |
| Cermak 2012 [14]  (N=26) | Beetroot juice (140ml) – once daily for 6 days.  Final dose 2.5 hours pre-exercise test. | Placebo juice (140ml) – once daily for 6 days.  Final dose 2.5 hours pre-exercise test. | None. | Male adults (18 yrs or older); Trained cyclists or triathletes;  31 (3) | Diastolic Blood Pressure, VO2, Plasma Nitrate, Heart Rate, Respiratory Exchange Ratio, Rating of Perceived Exertion, VCO2, Power Output, Systolic Blood Pressure, Time Trial Performance |
| Christensen 2013 [15]  (N=20) | Beetroot juice (500ml) for 7 days.  Final dose 3 hours pre-exercise test. | Apple/black currant juice (500ml) for 7 days.  Final dose 3 hours pre-exercise test. | None. | Male adults (18 yrs or older);  Elite cyclists competing at the highest domestic level;  29 (4) | VO2, Plasma Nitrate, Power Output, Time Trial Performance |
| Crum 2017 [16]  (N=16) | Pomegranate extract capsule (1000 mg) –2.5 hours pre-exercise test. | Placebo capsule – 2.5 hours pre-exercise test. | None. | Adults (18 yrs or older);  Current or former national cycling or triathlon development programmes;  33 (11) | Heart Rate, Blood Lactate, Time to Exhaustion, VO2, VCO2, |
| Crum 2018 [17]  (N=16) | Pomegranate extract capsules (15 mg/kg/day) for 8 days.  Final supplement 2.5 hours pre-exercise test. | Placebo capsule for 8 days.  Final supplement 2.5 hours pre-exercise test. | N-acetylcysteine capsule for 8 days. | Adults (18 yrs or older);  Trained cyclists from regional cycling community;  37 (11) | Power Output |
| De Castro 2018 [18]  (N=28) | Beetroot juice (420ml) – once daily for 3 days. | Nitrate-depleted beetroot juice (420ml) – once daily for 3 days. | None. | Adults (18 yrs or older);  Recreationally active;  30.1 (5.7) | Blood Lactate, Heart Rate, Rating of Perceived Exertion, Time Trial Performance |
| Esen 2018  (N=16) [19] | Beetroot juice (70ml) – twice a day for 3 days.  Final dose 3 hours pre-exercise test. | Nitrate-depleted beetroot juice (70ml) – twice a day for 3 days.  Final dose 3 hours pre-exercise test. | None. | Adults (18 yrs or older);  Moderately-trained swimmers;  22 (6) | Diastolic Blood Pressure, Systolic Blood Pressure, Time Trial Performance, Plasma Nitrite |
| Fan 2018 [20]  (N=24) | Sodium nitrate capsules (0.1 mmol/kg/day) for 8 days. | Sodium chloride  Capsules (0.1 mmol/kg/day) for 8 days. | None. | Male adults (18 yrs or older);  Trained cyclists;  31 (7) | Heart Rate, Ventilation |
| Garnacho-Castano 2018 [21]  (N=24) | Beetroot juice (70 mL) – one dose 3 hours pre-exercise test. | Nitrate-depleted beetroot juice (70ml) – one dose 3 hours pre-exercise test. | None. | Adults (18 yrs or older);  National and international triathletes;  39.3 (7.5) | VO2, Heart Rate, Respiratory Exchange Ratio, Ventilation, VCO2 |
| Glaister 2015 [22]  (N=28) | Beetroot juice (70ml) – one dose 2.5 hours pre-exercise test. | Nitrate-depleted beetroot juice (70ml) – one dose 2.5 hours pre-exercise test. | Single dose of 5 mg/kg of caffeine or maltodextrin (placebo). | Adults (18 yrs or older);  Triathletes and cyclists;  31 (7) | Time Trial Performance, Rating of Perceived Exertion, Plasma Nitrate, VO2, Plasma Nitrite, VO2, Plasma Nitrate, Rating of Perceived Exertion |
| Handzlik 2013 [23]  (N=28) | Beet root juice (70 mL) – two doses 150- and 75-min pre-exercise test. | Nitrate-depleted beetroot juice  (70ml) – two doses 150- and 75-min pre-exercise test. | Single dose of 5mg/kg caffeine provided 1-2 hours before exercise. | Adults (18 yrs or older);  Well-trained cyclists;  31 (7) | Time to Exhaustion, VO2, Heart Rate, Respiratory Exchange Ratio, Rating of Perceived Exertion, VCO2, |
| Hoon 2014 [24]  (N=56) | Beetroot concentrate (70ml) – two doses 150- and 75-min before first time trial and an additional half dose 75-min prior to second time trial. | Nitrate-depleted beetroot juice (70ml) – two doses 150- and 75-min before first time trial and an additional half dose 75 min prior to second time trial. | None. | Male adults (18 yrs or older);  Trained cyclists;  20.3 (1.4) | Power Output |
| Horiuchi 2017 [25]  (N=18) | Beetroot juice (140ml) – once daily for 3 days. | Nitrate-depleted beetroot juice (140ml) – once daily for 3 days. | None. | Adults (18 yrs or older);  Regular physical activity (1–2 hours per day, 3–5 days per week);  21 (3) | Blood Lactate, Time to Exhaustion, VO2, Plasma Nitrate, Mean Arterial Pressure, Heart Rate, Respiratory Exchange Ratio, VCO2, Ventilation |
| Jo 2017 [26]  (N=28) | Multi-day supplementation: One nitrate tablet (8 mmol) – once a day for 2 weeks.  Single-day supplementation: One nitrate tablet (8mmol) – 2.5 hours before testing. | Multi-day supplementation: One placebo tablet – once a day for 2 weeks.  Single-day supplementation: One placebo tablet – 2.5 hours before testing. | None. | Adults (18–29 years);  Recreationally active (within the past 6 months, at least 3 hours/week of aerobic exercise;  Multi-day Group: 23.43 (2.03)  Single-day Group: 22.20 (2.48) | Heart Rate, Power Output, Time Trial Performance |
| Kelly 2013 [27]  (N=18) | Beetroot juice (250 mL) – twice a day for 3 days before each test. | Nitrate-depleted beetroot juice (250ml) – twice a day for 3 days before each test. | None. | Male adults (18 yrs or older);  Recreationally active;  22 (3) | Resting Metabolic Rate, Work Done, Diastolic Blood Pressure, Systolic Blood Pressure, Mean Arterial Pressure, Plasma Nitrite, VO2, Time to Exhaustion, Power Output |
| Kelly 2014 [28]  (N=24) | Beetroot juice (70ml) – twice a day for 3 days. | Nitrate-depleted beetroot juice (70ml) – twice a day for 3 days. | None. | Adults (18 yrs or older);  Recreationally active;  22 (4) | Time to Exhaustion, VO2, Heart Rate, Plasma Nitrite |
| Kent 2018[29]  (N=12) | Beetroot juice (70ml) – once a day for 2 days. 140 ml on third day. | Nitrate-depleted beetroot juice (70ml) – once a day for 2 days. 140ml on third day. | None. | Male adults (18 yrs or older);  Endurance-trained cyclists  26.6 (4.4) | Plasma Nitrate, Heart Rate, Rating of Perceived Exertion, Plasma Nitrite |
| Kent 2018 [30]  (N=12) | Beetroot juice (70ml) – once a day for 2 days. 140 mL on third day. | Nitrate-depleted beetroot juice (70ml) – once a day for 2 days. 140 mL on third day. | None. | Adults (18 yrs or older);  Athletes from local cycling and triathlon clubs;  27 (6) | Plasma Nitrate, Time Trial Performance, Plasma Nitrite |
| Kramer 2016 [31]  (N=24) | Nitrate capsule (4mmol) – twice a day for 6 days. | Placebo capsule (4mmol) – twice a day for 6 days. | None. | Male adults (18 yrs or older);  CrossFit athletes;  23 (5) | Power Output, Time Trial Performance |
| Lane 2014 [32]  (N=48) | Beetjuice shots (140mL) – two doses (8 to 12 hours pre-exercise test and second dose 130 minutes pre-exercise test). | Nitrate-depleted beetjuice (140ml) – two doses (8 to 12 hours pre-exercise test and second dose 130 minutes pre-exercise test). | None. | Adults (18 yrs or older);  Competitive cyclists;  31 (7) | Heart Rate, Rating of Perceived Exertion, Power Output, Time Trial Performance |
| Lansley 2011 [33]  (N=18) | Beetroot juice (500ml) – single dose 2.5 hours pre-exercise test. | Nitrate-depleted beetroot juice (500ml) – single 2.5 hours pre-exercise test. | None. | Male adults (18 yrs or older);  Competitive cyclists;  21 (4) | Blood Lactate, Diastolic Blood Pressure, Time to Exhaustion, VO2, Mean Arterial Pressure, Heart Rate, Respiratory Exchange Ratio, Ventilation |
| Larsen 2007 [34]  (N=18) | Sodium nitrate – 0.1 mmol sodium nitrate/kg – three times a day for 3 days. | Sodium chloride – three times a day for 3 days. | None. | Adults (18 yrs or older);  Trained cyclists or triathletes;  28 (6) | Diastolic Blood Pressure, VO2, Plasma Nitrate, Heart Rate, Ventilation, Power Output, Systolic Blood Pressure, Plasma Nitrite |
| Larsen 2011 [35]  (N=28) | Sodium nitrate – 0.1 mmol/kg/day – three times a day for 3 days. | Sodium chloride – three times a day for 3 days. | None. | Healthy, non-smoking adults (18 yrs or older);  25 (1) | VO2, Plasma Nitrate, Respiratory Exchange Ratio, Plasma Nitrite |
| Lowings 2017 [36]  (N=20) | Beetroot juice (140ml). | Nitrate-depleted beetroot juice (140ml). | None. | Adults (18 yrs or older);  University students trained minimum of 3 times per week, and competed locally and for their university;  20 (1) | Blood Lactate, Exhaled NO, Time Trial Performance |
| Masschelein 2012 [37]  (N=30) | Beetroot juice (100ml) – once a day for 5 days. | Apple-black currant juice (100ml) – once a day for 5 days. | None. | Adults (18 yrs or older);  Recreationally active;  21.1 (1) | VO2, Plasma Nitrate, Heart Rate, Respiratory Exchange Ratio, Ventilation, Rating of Perceived Exertion, VCO2, Time to Exhaustion, Plasma Nitrite |
| McQuillan 2017 [38]  (N=18) | Beetroot juice (70ml) – once daily for 6-8 days. | Placebo juice (70mL) – once daily for 6-8 days. | None. | Male adults (18 yrs or older);  Endurance trained cyclists;  26 (8) | VO2, Heart Rate, Ventilation, Power Output, Time Trial Performance |
| McQuillan 2018 [39]  (N=16) | Beetroot juice (140ml) – once daily for 3 days. | Placebo juice (140ml) – once daily for 3 days. | None. | Male adults (18 yrs or older);  Well-trained competitive endurance cyclists;  25 (8) | Power Output, Plasma Nitrite |
| Meamarbashi 2014 [40]  (N=33) | Group 1 – watercress (100g) for 7 days.  Group 2 – red radish (100g) for 7 days. | No consumption of high nitrate foods for 7 days. | None. | Male adults (18 yrs or older);  Healthy and non-active university students;  Group 1: 20.2 (1.62)  Group 2: 20.5 (2.25) | Diastolic Blood Pressure, VO2, Heart Rate, Respiratory Exchange Ratio, Ventilation, Work Done, VCO2, Distance Travelled, Power Output |
| Muggeridge 2013 [41]  (N=16) | Beetroot juice (70ml) – one dose 3 hours pre-exercise test. | Tomato juice (70ml) – one dose 3 hours pre-exercise test. | None. | Male adults (18 yrs or older);  Trained paddlers recruited from local paddle sport clubs;  31 (15) | Diastolic Blood Pressure, VO2, Plasma Nitrate, Heart Rate, Power Output, Systolic Blood Pressure, Time Trial Performance, Plasma Nitrite |
| Muggeridge 2014 (1) [42]    (N=18) | Nitrate-rich gel with extracts of Swiss chard and Rhubarb (120ml) – once 2.5 hours pre-exercise test. | Placebo gel (120ml) – once 2.5 hours pre-exercise test. | Ultraviolet (UV)-A light. | Male adults (18 yrs or older);  Trained cyclists and triathletes;  36 (6) | Diastolic Blood Pressure, VO2, Mean Arterial Pressure, Heart Rate, Systolic Blood Pressure, Time Trial Performance, Diastolic Blood Pressure |
| Muggeridge 2014 (2) [43]  (N=18) | Beetroot juice (70ml). | Placebo juice (70ml). | None. | Male adults (18 yrs or older);  Trained cyclists recruited from local cycling and triathlon clubs;  28 (8) | Diastolic Blood Pressure, Plasma Nitrate, Mean Arterial Pressure, Power Output, Systolic Blood Pressure, Time Trial Performance Plasma Nitrite |
| Mumford 2018 [44]  (N=56) | Beetroot concentrate (50mg) – twice a day for 6 days. | Placebo (50mg) – twice a day for 6 days. | None. | Adults (19 to 50 yrs of age) who were not currently consuming beet roots, free of any cardiovascular, respiratory, metabolic, or musculoskeletal disease, and not taking any other supplements or ergogenic aids;  Cycling 3–7 days per week and totaling a weekly mileage of greater than 100 miles;  29 (10) | Diastolic Blood Pressure, Heart Rate, Power Output, Systolic Blood Pressure |
| Nyback 2017 [45]  (N=16) | Beetroot juice (140ml) – once 2.5 hours pre-exercise test. | Placebo juice (140ml) – once 2.5 hours pre-exercise test. | None. | Adults (18 yrs or older);  Well-trained cross-country skiers competing at a national level;  Males: 21.8 (2.8)  Females: 20.7 (1.2) | Blood Lactate, Diastolic Blood Pressure, VO2, Plasma Nitrate, Heart Rate, Respiratory Exchange Ratio, Rating of Perceived Exertion |
| Peacock 2012 [46]  (N=20) | Potassium nitrate capsule (1 g) – once 2.5 hours pre-exercise test. | Placebo capsule – once 2.5 hours pre-exercise test. | None. | Male adults (18 yrs of age);  Norwegian junior-elite cross-country skiers;  18 (0) | Blood Lactate, Oxygen Uptake, Plasma Nitrate, Heart Rate, Respiratory Exchange Ratio, Time Trial Performance, Plasma Nitrite |
| Porcelli 2015 [47]  (N=42) | Sodium nitrate (5.5 mmol) – once daily for 6 days. | Sodium chloride (8.0 mmol) – once daily for 6 days. | None. | Adults (18 yrs or older);  Varying levels of sports;  22.7 (1.8) | Blood Lactate, VO2, Plasma Nitrate, Speed, Heart Rate, Respiratory Exchange Ratio, Ventilation, Rating of Perceived Exertion, VCO2 |
| Porcelli 2016 [48]  (N=14) | High nitrate diet (8.2 mmol/day) for 6 days. | Control diet (2.9 mmol/day) for 6 days. | None. | Male adults (18 yrs or older);  Recreationally involved in basketball, badminton, and futsal;  25 (2) | Blood Lactate, VO2, Plasma Nitrate, Heart Rate, Respiratory Exchange Ratio, Ventilation, VCO2, Power Output, Plasma Nitrite |
| Pryor 2017 [49]  (N=20) | Betaine (2.5g) in carbohydrate-electrolyte beverage (250ml) – once 45 minutes pre-exercise test. | Placebo in carbohydrate-electrolyte beverage (250ml) – once 45 minutes pre-exercise test. | None. | Adults (18 yrs or older);  Recreationally active;  20.2 (3.6) | Plasma Nitrate |
| Puype 2015 [50]  (N=44) | Beetroot juice (700 mg of nitrate/L) – three times a day for 4 days. | Apple–blackcurrant juice – three times a day for 4 days. | None. | Male adults (18–30 yrs of age);  Recreationally active;  Nitrate: 21.7 (0.6)  Placebo: 21.4 (0.8) | Blood Lactate, Time to Exhaustion, VO2, Heart Rate, Respiratory Exchange Ratio, Power Output |
| Rienks 2015 [51]  (N=20) | Beetroot juice (140ml) – once 2.5 hours pre-exercise test. | Placebo juice (140mL) – once 2.5 hours pre-exercise test. | None. | Adults (18 yrs or older);  Regular exercise, but not competitive;  25 (3) | Diastolic Blood Pressure, VO2, Mean Arterial Pressure, Heart Rate, Respiratory Exchange Ratio, Ventilation, Work Done, VCO2, Power Output, Systolic Blood Pressure |
| Rimer 2016 [52]  (N=26) | Beetroot juice (140ml). | Nitrate-depleted beetroot juice (140ml). | None. | Adults (18 yrs or older);  Competitively trained athletes (NCAA & national-level athletes);  25.9 (7.5) | Optimal Pedalling Rate, Power Output |
| Rossetti 2017 [53]  (N=40) | Beetroot juice (70ml) – once daily for 6 days. | Placebo juice (70ml) – once daily for 6 days. | None. | Male adults (18 yrs or older);  Recreationally active  25 (3) | Diastolic Blood Pressure, VO2, Mean Arterial Pressure, Heart Rate, Respiratory Exchange Ratio, Ventilation, Systolic Blood Pressure |
| Sandbakk 2015 [54]  (N=18) | Potassium nitrate capsule (1g) – once 2.5 hours pre-exercise test. | Placebo capsule – once 2.5 hours pre-exercise test. | L-arginine capsule (1g) – once 2.5 hours prior to testing. | Male adults (18 yrs of age);  Norwegian junior-elite cross-country skiers;  18 (0) | Blood Lactate, VO2, Plasma Nitrate, Mean Arterial Pressure, Respiratory Exchange Ratio, Ventilation, Time Trial Performance, Plasma Nitrite, VO2, Plasma Nitrate, Respiratory Exchange Ratio, Plasma Nitrite, Mean Arterial Pressure, Ventilation |
| Shannon 2017 [55]  (N=16) | Beetroot juice (40ml) – once 3 hours pre-exercise test. | Placebo juice (40ml) – once 3 hours pre-exercise test. | None. | Male adults (18 yrs or older);  Trained runners or triathletes;  28.3 (5.8) | VO2, Mean Arterial Pressure, Heart Rate, Respiratory Exchange Ratio, Rating of Perceived Exertion, VCO2 |
| Shannon 2016 [56]  (N=24) | Beetroot juice (138ml) – once 3 hours pre-exercise test. | Placebo juice (138ml) – once 3 hours pre-exercise test. | None. | Adults (18 yrs or older);  Varying levels of fitness;  24.4 (3) | VO2, Plasma Nitrate, Exhaled NO, Mean Arterial Pressure, Heart Rate, Rating of Perceived Exertion, Time Trial Performance, Plasma Nitrite |
| Trexler 2014 [57]  (N=38) | Pomegranate extract (1g) – once 30 minutes pre-exercise test. | Placebo – once 30 minutes pre-exercise test. | None. | Adults (18 yrs or older);  Regular exercise program for at least 2 months prior to the study;  22.2 (2.2) | Time to Exhaustion |
| Vanhatalo 2010 [58]  (N=16) | Beetroot juice (500ml) – once daily for 15 days. | Placebo juice (500ml) – once daily for 15 days. | None. | Adults (18 yrs or older);  Recreationally active;  29 (6) | Power Output, Heart Rate, Blood Lactate, VO2, Respiratory Exchange Ratio, VCO2 |
| Vanhatalo 2011 [59]  (N=18) | Beetroot juice (250ml) – three doses 24 hours, 12 hours and 2.5 hours pre-exercise test. | Nitrate-depleted beetroot juice (250ml) – three doses 24 hours, 12 hours and 2.5 hours pre-exercise test. | None. | Adults (18 yrs or older);  Moderately trained in recreational sport;  28 (7) | Diastolic Blood Pressure, Time to Exhaustion, Mean Arterial Pressure, Heart Rate, Systolic Blood Pressure, Plasma Nitrite |
| Van Hoorebeke 2016 [60]  (N=30) | Beetroot concentrate capsule (50 mg) – once 140 minutes pre-exercise test. | Placebo capsule – once 140 minutes pre-exercise test. | None. | Male adults (18 yrs or older);  Recreationally-competitive runners;  25.3 (5.4) | Blood Lactate, VO2, Heart Rate, Respiratory Exchange Ratio, Rating of Perceived Exertion |
| Wilkerson 2012 [61]  (N=16) | Beetroot juice (500ml) – once 2.5 hours pre-exercise test. | Nitrate-depleted beetroot juice (500ml) – once 2.5 hours pre-exercise test. | None. | Male adults (18 yrs or older); Well-trained cyclists;  31 (11) | 50-Mile Time Trial Performance |
| Wylie 2016 [62]  (N=20) | Beetroot juice (70ml) – twice a day for 5 days. | Nitrate-depleted beetroot juice (70ml) – twice a day for 5 days. | None. | Male adults (18 yrs or older);  Recreational team-sport players;  21 (1.1) | Plasma NO, Power Output, Blood Lactate, VO2, VCO2, Respiratory Exchange Ratio |
| Hurst 2020 [63]  (N=70) | Beetroot juice (70ml) – one dose.  2.5 hours pre-exercise test. | Nitrate-depleted beetroot juice (70ml) – 2.5 hours pre-exercise test. | None. | Adults (18 years or older); Recreational runners; 33.3 (12.3) | 5-Km Time Trial Performance |
| Berry 2020 [64]  (N=30) | High-nitrate beverage (four ounces; 9.9 mmols of nitrate) – once a day for 7 days. | Low-nitrate beverage (four ounces; 0.5 mmoles of nitrate) – once a day for 7 days. | None. | Adults (41-64 years old); Well-trained middle to older-aged adults; 49 (6) | Plasma NO2, Plasma NO3, Time Trial Performance, Power Output, VO2, Systolic Blood Pressure, Diastolic Blood Pressure, Heart Rate, Rating of Perceived Exertion |
| Gholami 2019 [65]  (N=20) | 8, 16, or 24 mmols of sodium nitrate dissolved in water – one dose. 2.5 hours pre-exercise test. | NaCl dissolved in water – one dose. 2.5 hours pre-exercise test. | None. | Male adults; Well-trained; 22.62 (3) | Serum NO; Plasma Peroxynitrite; Plasma Lactate; Time to Exhaustion; Rating of Perceived Exertion; VO2; Respiratory Exchange Ratio; Gastrointestinal Discomfort |
| Torregrosa-Garcia 2019 [66] (N=52) | Pomegranate extract capsule (375 mg) – two capsules per day for 15 days. | Maltodextrin capsule – two capsules per day for 15 days. | None. | Male adults; Amateur cyclists; 34.9 (10) | Rating of Perceived Exertion; Time to Exhaustion; Time to Reach Ventilatory Threshold 2; VO2max; VO2 at Ventilatory Threshold 2; Blood Lactate |
| Cocksedge 2020 [67] (N=20) | Beetroot juice (210ml) – one dose. 2.5 hours pre-exercise test. | Nitrate-depleted beetroot juice (210ml) – one dose. 2.5 hours pre-exercise test. | None. | Male adults; Recreationally active; 23 (3) | Plasma Nitrite; Arterial Oxygen Saturation; Tissue Oxygenation Index; Pulmonary Oxygen Uptake; Exercise Tolerance |
| Santana 2019 [68] (N=16) | Nitrate capsule (750 mg of inorganic nitrate and 5 g of resistant starch) – once per day for 30 days. | Nitrate capsule (6 g of resistant starch) – once per day for 30 days. | None | Male adults; Recreationally active; 30.15 (5.65) | 10-Km Time Trial Performance, Power Output, Blood Lactate |
| Robinson 2021 [69] (N=16) | Beetroot juice (140 ml) – 2 X 70ml daily for 7 days. 3 hours pre-exercise test. | Nitrate-depleted beetroot juice (140 ml) – 2 X 70ml daily for 7 days. 3 hours pre-exercise test. | None. | Male adults; Endurance-trained recreational athletes; 23 (4) | SpO2; Plasma Nitrite; Time to Exhaustion |
| Wickham 2018 [70] (N=24) | Acute: Beetroot juice (280ml) – one dose. 2.5 hours pre-exercise test.  Chronic: Beetroot juice (280ml) – once per day for 8 days. | Acute: Nitrate-depleted beetroot juice (280ml) – one dose. 2.5 hours pre-exercise test.  Chronic: Nitrate-depleted beetroot juice (280ml) – once per day for 8 days. | None. | Female adults; Recreationally active; 23 (1) | VO2; Time Trial Performance |
| Perez 2019 [71] (N=40) | Beetroot juice (70ml) – once per day for 7 days. | Nitrate-depleted beetroot juice (237ml) – once per day for 7 days. | None. | Adults; Recreationally active; 21.8 (2.35) | VO2max; Systolic Blood Pressure; Diastolic Blood Pressure; Respiratory Exchange Ratio; Rating of Perceived Exertion |
| Rokkedal-Lausch 2019 [72] (N=24) | Beetroot juice (140ml) – once per day for 7 days. | Nitrate-depleted beetroot juice (140ml) – once per day for 7 days. | None. | Male adults; Well-trained male cyclists; 29.1 (7.7) | Power Output; Time Trial Performance; VO2; VCO2; Ventilation; Respiratory Exchange Ratio; Heart Rate; SpO2; VO2peak; Heart Rate Peak; Respiratory Exchange Ratio Peak; Oxygenated Hemoglobin; Deoxygenated Hemoglobin; Total Hemoglobin |
| Casado 2021 [73] (N=48) | Beetroot juice (140ml) – one dose. 2.5 hours pre-exercise test. | Nitrate-depleted beetroot juice (140ml) – one dose. 2.5 hours pre-exercise test. | None. | Adults; Well-trained amateur runners; Males: 38.7 (9.2); Females: 36.6 (8.2) | 2-km Time Trial Performance; Blood Lactate; Rating of Perceived Exertion |

1. Amano T, Okushima D, Breese B, Bailey S, Koga S, Kondo N. Influence of dietary nitrate supplementation on local sweating and cutaneous vascular responses during exercise in a hot environment. European Journal of Applied Physiology. 2018 08/01;118.

2. Arnold J, Oliver S, Lewis-Jones T, Wylie L, Macdonald J. Beetroot Juice Does Not Enhance Altitude Running Performance in Well-Trained Athletes. Applied Physiology Nutrition and Metabolism. 2015 02/04;40:2015.

3. Aucouturier J, Boissière J, Pawlak-Chaouch M, Cuvelier G, Gamelin F-X. Effect of dietary nitrate supplementation on tolerance to supramaximal intensity intermittent exercise. Nitric oxide : biology and chemistry / official journal of the Nitric Oxide Society. 2015 05/28;49.

4. Bailey SJ, Winyard P, Vanhatalo A, Blackwell JR, DiMenna FJ, Wilkerson DP, et al. Dietary nitrate supplementation reduces the O2 cost of low-intensity exercise and enhances tolerance to high-intensity exercise in humans. Journal of Applied Physiology. 2009;107(4):1144-55.

5. Bailey SJ, Varnham RL, DiMenna FJ, Breese BC, Wylie LJ, Jones AM. Inorganic nitrate supplementation improves muscle oxygenation, O2 uptake kinetics, and exercise tolerance at high but not low pedal rates. Journal of Applied Physiology. 2015;118(11):1396-405.

6. Balsalobre-Fernández C, Romero-Moraleda B, Cupeiro R, Peinado A, Butragueño J, Benito PJ. The effects of beetroot juice supplementation on exercise economy, rating of perceived exertion and running mechanics in elite distance runners: A double-blinded, randomized study. PLOS ONE. 2018 07/11;13:e0200517.

7. Bescós R, Rodríguez F, Iglesias X, Ferrer M, Iborra E, Pons A. Acute Administration of Inorganic Nitrate Reduces V˙O2peak in Endurance Athletes. Medicine and science in sports and exercise. 2011 03/01;43:1979-86.

8. Betteridge S, Bescós R, Martorell M, Pons A, Garnham A, Stathis C, et al. No effect of acute beetroot juice ingestion on oxygen consumption, glucose kinetics or skeletal muscle metabolism during submaximal exercise in males. Journal of Applied Physiology. 2015 12/03;120:jap.00658.2015.

9. Bourdillon N, Fan J-L, Uva B, Müller H, Meyer P, Kayser B. Effect of oral nitrate supplementation on pulmonary hemodynamics during exercise and time trial performance in normoxia and hypoxia: a randomized controlled trial. Frontiers in physiology. 2015;6:288-.

10. Breese BC, McNarry MA, Marwood S, Blackwell JR, Bailey SJ, Jones AM. Beetroot juice supplementation speeds O2 uptake kinetics and improves exercise tolerance during severe-intensity exercise initiated from an elevated metabolic rate. American Journal of Physiology-Regulatory, Integrative and Comparative Physiology. 2013;305(12):R1441-R50.

11. Breese B, Poole D, Okushima D, Bailey S, Jones A, Kondo N, et al. The effect of dietary nitrate supplementation on the spatial heterogeneity of quadriceps deoxygenation during heavy‐intensity cycling. Physiological Reports. 2017 07/01;5.

12. Callahan MJ, Parr EB, Hawley J, Burke LM. Single and Combined Effects of Beetroot Crystals and Sodium Bicarbonate on 4-km Cycling Time Trial Performance. International journal of sport nutrition and exercise metabolism. 2017;27 3:271-8.

13. Cermak NM, Res P, Stinkens R, Lundberg JO, Gibala MJ, Loon LJCv. No Improvement in Endurance Performance after a Single Dose of Beetroot Juice. 2012;22(6):470.

14. Cermak NM, Gibala MJ, Loon LJCv. Nitrate Supplementation’s Improvement of 10-km Time-Trial Performance in Trained Cyclists. 2012;22(1):64.

15. Christensen PM, Nyberg M, Bangsbo J. Influence of nitrate supplementation on VO2 kinetics and endurance of elite cyclists. Scandinavian Journal of Medicine & Science in Sports. 2013;23(1):e21-e31.

16. Crum EM, Che Muhamed AM, Barnes M, Stannard SR. The effect of acute pomegranate extract supplementation on oxygen uptake in highly-trained cyclists during high-intensity exercise in a high altitude environment. Journal of the International Society of Sports Nutrition. 2017 2017/05/31;14(1):14.

17. Crum EM, Barnes MJ, Stannard SR. Multiday Pomegranate Extract Supplementation Decreases Oxygen Uptake During Submaximal Cycling Exercise, but Cosupplementation With N-acetylcysteine Negates the Effect. 2018;28(6):586.

18. Castro TFd, Manoel F, Figueiredo D, Figueiredo DH, Machado FA. Effect of beetroot juice supplementation on 10-km performance in recreational runners. Applied physiology, nutrition, and metabolism = Physiologie appliquee, nutrition et metabolisme. 2019;44 1:90-4.

19. Esen O, Nicholas C, Morris M, Bailey S. No Effect of Beetroot Juice Supplementation on 100-m and 200-m Swimming Performance in Moderately-Trained Swimmers. International Journal of Sports Physiology and Performance. 2018 11/14;14:1-19.

20. Fan J-L, Bourdillon N, Meyer P, Kayser B. Oral Nitrate Supplementation Differentially Modulates Cerebral Artery Blood Velocity and Prefrontal Tissue Oxygenation During 15 km Time-Trial Cycling in Normoxia but Not in Hypoxia. Frontiers in physiology. 2018;9:869-.

21. Garnacho-Castaño MV, Palau-Salvà G, Cuenca E, Muñoz-González A, García-Fernández P, del Carmen Lozano-Estevan M, et al. Effects of a single dose of beetroot juice on cycling time trial performance at ventilatory thresholds intensity in male triathletes. Journal of the International Society of Sports Nutrition. 2018 2018/10/04;15(1):49.

22. Glaister M, Pattison J, Muniz D, Patterson S, Foley P. Effects of Dietary Nitrate, Caffeine, and Their Combination on 20-km Cycling Time Trial Performance. Journal of strength and conditioning research / National Strength & Conditioning Association. 2014 06/24;29.

23. Handzlik MK, Gleeson M. Likely Additive Ergogenic Effects of Combined Preexercise Dietary Nitrate and Caffeine Ingestion in Trained Cyclists. ISRN Nutrition. 2013 2013/12/14;2013:396581.

24. Hoon MW, Hopkins WG, Jones AM, Martin DT, Halson SL, West NP, et al. Nitrate supplementation and high-intensity performance in competitive cyclists. Applied Physiology, Nutrition, and Metabolism. 2014 2014/09/01;39(9):1043-9.

25. Horiuchi M, Endo J, Dobashi S, Handa Y, Kiuchi M, Koyama K. Muscle oxygenation profiles between active and inactive muscles with nitrate supplementation under hypoxic exercise. Physiological reports. 2017;5(20):e13475.

26. Jo E, Fischer M, Auslander A, Beigarten A, Daggy B, Hansen K, et al. The Effects of Multi-Day vs. Single Pre-exercise Nitrate Supplement Dosing on Simulated Cycling Time Trial Performance and Skeletal Muscle Oxygenation. Journal of Strength and Conditioning Research. 2017 04/01;33:1.

27. Kelly J, Vanhatalo A, Wilkerson D, Wylie L, Jones A. Effects of Nitrate on the Power–Duration Relationship for Severe-Intensity Exercise. Medicine and science in sports and exercise. 2013 03/07;45.

28. Kelly J, Vanhatalo A, Bailey SJ, Wylie LJ, Tucker C, List S, et al. Dietary nitrate supplementation: effects on plasma nitrite and pulmonary O2 uptake dynamics during exercise in hypoxia and normoxia. American Journal of Physiology-Regulatory, Integrative and Comparative Physiology. 2014;307(7):R920-R30.

29. Kent GL, Dawson B, Cox GR, Abbiss CR, Smith KJ, Croft KD, et al. Effect of dietary nitrate supplementation on thermoregulatory and cardiovascular responses to submaximal cycling in the heat. European Journal of Applied Physiology. 2018 2018/03/01;118(3):657-68.

30. Kent GL, Dawson B, Cox GR, Burke LM, Eastwood A, Croft KD, et al. Dietary nitrate supplementation does not improve cycling time-trial performance in the heat. Journal of Sports Sciences. 2018 2018/06/03;36(11):1204-11.

31. Kramer SJ, Baur DA, Spicer MT, Vukovich MD, Ormsbee MJ. The effect of six days of dietary nitrate supplementation on performance in trained CrossFit athletes. Journal of the International Society of Sports Nutrition. 2016 2016/11/03;13(1):39.

32. Lane SC, Hawley JA, Desbrow B, Jones AM, Blackwell JR, Ross ML, et al. Single and combined effects of beetroot juice and caffeine supplementation on cycling time trial performance. Applied Physiology, Nutrition, and Metabolism. 2013 2014/09/01;39(9):1050-7.

33. Lansley K, Winyard P, Bailey S, Vanhatalo A, Wilkerson D, Blackwell J, et al. Acute Dietary Nitrate Supplementation Improves Cycling Time Trial Performance. Medicine and science in sports and exercise. 2011 04/01;43:1125-31.

34. Larsen F, Weitzberg E, Lundberg JO, Ekblom B. Effect of dietary nitrate on oxygen cost during exercise. Acta physiologica (Oxford, England). 2007 10/01;191:59-66.

35. Larsen FJ, Schiffer TA, Borniquel S, Sahlin K, Ekblom B, Lundberg JO, et al. Dietary Inorganic Nitrate Improves Mitochondrial Efficiency in Humans. Cell Metabolism. 2011 2011/02/02/;13(2):149-59.

36. Lowings S, Shannon OM, Deighton K, Matu J, Barlow MJ. Effect of Dietary Nitrate Supplementation on Swimming Performance in Trained Swimmers. 2017;27(4):377.

37. Masschelein E, Thienen RV, Wang X, Schepdael AV, Thomis M, Hespel P. Dietary nitrate improves muscle but not cerebral oxygenation status during exercise in hypoxia. Journal of Applied Physiology. 2012;113(5):736-45.

38. McQuillan JA, Dulson DK, Laursen PB, Kilding AE. The Effect of Dietary Nitrate Supplementation on Physiology and Performance in Trained Cyclists. 2017;12(5):684.

39. McQuillan JA, Casadio JR, Dulson DK, Laursen PB, Kilding AE. The Effect of Nitrate Supplementation on Cycling Performance in the Heat in Well-Trained Cyclists. 2018;13(1):50.

40. Meamarbashi A, Alipour M. Moderate dose of watercress and red radish does not reduce oxygen consumption during graded exhaustive exercise. Avicenna J Phytomed. 2014;4(4):267-72.

41. Muggeridge D, Howe C, Spendiff O, Pedlar C, James P, Easton C. The Effects of a Single Dose of Concentrated Beetroot Juice on Performance in Trained Flatwater Kayakers. International journal of sport nutrition and exercise metabolism. 2013 04/09;23.

42. Muggeridge D, Sculthorpe N, Garce F, Ratcliffe J, Weller R, James P, et al. Acute Whole-Body UVA Irradiation Combined with Nitrate Ingestion Enhances Cycling Performance in Trained Cyclists; 2014.

43. Muggeridge D, Howe C, Spendiff O, Pedlar C, James P, Easton C. A Single Dose of Beetroot Juice Enhances Cycling Performance in Simulated Altitude. Medicine and science in sports and exercise. 2013 07/10;46.

44. Mumford PW, Kephart WC, Romero MA, Haun CT, Mobley CB, Osburn SC, et al. Effect of 1-week betalain-rich beetroot concentrate supplementation on cycling performance and select physiological parameters. European Journal of Applied Physiology. 2018 2018/11/01;118(11):2465-76.

45. Nybäck L, Glännerud C, Larsson G, Weitzberg E, Shannon O, McGawley K. Physiological and performance effects of nitrate supplementation during roller-skiing in normoxia and normobaric hypoxia. Nitric Oxide. 2017 08/01;70.

46. Peacock O, Tjønna A, James P, Wisloff U, Welde B, Böhlke N, et al. Dietary Nitrate Does Not Enhance Running Performance in Elite Cross-Country Skiers. Medicine and science in sports and exercise. 2012 08/07;44:2213-9.

47. PORCELLI S, RAMAGLIA M, BELLISTRI G, PAVEI G, PUGLIESE L, MONTORSI M, et al. Aerobic Fitness Affects the Exercise Performance Responses to Nitrate Supplementation. Medicine & Science in Sports & Exercise. 2015;47(8):1643-51.

48. Porcelli S, Pugliese L, Rejc E, Pavei G, Bonato M, Montorsi M, et al. Effects of a Short-Term High-Nitrate Diet on Exercise Performance. Nutrients. 2016 08/31;8:534.

49. Pryor JL, Wolf S, Sforzo G, Swensen T. The Effect of Betaine on Nitrate and Cardiovascular Response to Exercise. International Journal of Exercise Science. 2017;10:550 - 9.

50. Puype J, Ramaekers M, Van Thienen R, Deldicque L, Hespel P. No effect of dietary nitrate supplementation on endurance training in hypoxia. Scandinavian Journal of Medicine & Science in Sports. 2015;25(2):234-41.

51. Rienks JN, Vanderwoude AA, Maas E, Blea ZM, Subudhi AW. Effect of Beetroot Juice on Moderate-Intensity Exercise at a Constant Rating of Perceived Exertion. International journal of exercise science. 2015;8(3):277-86.

52. Rimer EG, Peterson LR, Coggan AR, Martin JC. Increase in Maximal Cycling Power With Acute Dietary Nitrate Supplementation. 2016;11(6):715.

53. Rossetti GMK, Macdonald JH, Wylie LJ, Little SJ, Newton V, Wood B, et al. Dietary nitrate supplementation increases acute mountain sickness severity and sense of effort during hypoxic exercise. Journal of Applied Physiology. 2017;123(4):983-92.

54. Sandbakk S, Sandbakk O, Peacock O, James P, Welde B, Stokes K, et al. Effects of Acute Supplementation of L-arginine and Nitrate on Endurance and Sprint Performance in Elite Athletes. Nitric Oxide. 2014 10/22;48.

55. Shannon OM, Barlow MJ, Duckworth L, Williams E, Wort G, Woods D, et al. Dietary nitrate supplementation enhances short but not longer duration running time-trial performance. European Journal of Applied Physiology. 2017 2017/04/01;117(4):775-85.

56. Shannon O, Duckworth L, Barlow M, Woods D, Lara J, Siervo M, et al. Dietary nitrate supplementation enhances high-intensity running performance in moderate normobaric hypoxia, independent of aerobic fitness. Nitric Oxide. 2016 08/20;59:63-70.

57. Trexler ET, Smith-Ryan AE, Melvin MN, Roelofs EJ, Wingfield HL. Effects of pomegranate extract on blood flow and running time to exhaustion. Applied physiology, nutrition, and metabolism = Physiologie appliquee, nutrition et metabolisme. 2014;39(9):1038-42.

58. Vanhatalo A, Bailey SJ, Blackwell JR, DiMenna FJ, Pavey TG, Wilkerson DP, et al. Acute and chronic effects of dietary nitrate supplementation on blood pressure and the physiological responses to moderate-intensity and incremental exercise. American Journal of Physiology-Regulatory, Integrative and Comparative Physiology. 2010;299(4):R1121-R31.

59. Vanhatalo A, Fulford J, Bailey SJ, Blackwell JR, Winyard PG, Jones AM. Dietary nitrate reduces muscle metabolic perturbation and improves exercise tolerance in hypoxia. The Journal of physiology. 2011;589(Pt 22):5517-28.

60. Van Hoorebeke J, Trias C, Davis B, Lozada C, Casazza G. Betalain-Rich Concentrate Supplementation Improves Exercise Performance in Competitive Runners. Sports. 2016 07/25;4:40.

61. Wilkerson D, Hayward G, Bailey S, Vanhatalo A, Blackwell J, Jones A. Influence of acute dietary nitrate supplementation on 50 mile time trial performance in well-trained cyclists. European journal of applied physiology. 2012 04/20;112.

62. Wylie LJ, Bailey SJ, Kelly J, Blackwell JR, Vanhatalo A, Jones AM. Influence of beetroot juice supplementation on intermittent exercise performance. European journal of applied physiology. 2016;116(2):415-25.

63. Hurst P, Saunders S, Coleman D. No Differences Between Beetroot Juice and Placebo on Competitive 5-km Running Performance: A Double-Blind, Placebo-Controlled Trial. Int J Sport Nutr Exerc Metab. 2020 May 29;30(4):295-300.

64. Berry MJ, Miller GD, Kim-Shapiro DB, Fletcher MS, Jones CG, Gauthier ZD, et al. A randomized controlled trial of nitrate supplementation in well-trained middle and older-aged adults. PLoS One. 2020;15(6):e0235047.

65. Gholami F, Rahmani L, Amirnezhad F, Cheraghi K. High doses of sodium nitrate prior to exhaustive exercise increases plasma peroxynitrite levels in well-trained subjects: randomized, double-blinded, crossover study. Appl Physiol Nutr Metab. 2019 Dec;44(12):1305-10.

66. Torregrosa-Garcia A, Avila-Gandia V, Luque-Rubia AJ, Abellan-Ruiz MS, Querol-Calderon M, Lopez-Roman FJ. Pomegranate Extract Improves Maximal Performance of Trained Cyclists after an Exhausting Endurance Trial: A Randomised Controlled Trial. Nutrients. 2019 Mar 28;11(4).

67. Cocksedge SP, Breese BC, Morgan PT, Nogueira L, Thompson C, Wylie LJ, et al. Influence of muscle oxygenation and nitrate-rich beetroot juice supplementation on O2 uptake kinetics and exercise tolerance. Nitric Oxide. 2020 Jun 1;99:25-33.

68. Santana J, Madureira D, de Franca E, Rossi F, Rodrigues B, Fukushima A, et al. Nitrate Supplementation Combined with a Running Training Program Improved Time-Trial Performance in Recreationally Trained Runners. Sports (Basel). 2019 May 21;7(5).

69. Robinson GP, Killer SC, Stoyanov Z, Stephens H, Read L, James LJ, et al. Influence of Dietary Nitrate Supplementation on High-Intensity Intermittent Running Performance at Different Doses of Normobaric Hypoxia in Endurance-Trained Males. Int J Sport Nutr Exerc Metab. 2020 Dec 1;31(1):1-8.

70. Wickham KA, McCarthy DG, Pereira JM, Cervone DT, Verdijk LB, van Loon LJC, et al. No effect of beetroot juice supplementation on exercise economy and performance in recreationally active females despite increased torque production. Physiol Rep. 2019 Jan;7(2):e13982.

71. Perez JM, Dobson JL, Ryan GA, Riggs AJ. The Effects of Beetroot Juice on VO2max and Blood Pressure during Submaximal Exercise. Int J Exerc Sci. 2019;12(2):332-42.

72. Rokkedal-Lausch T, Franch J, Poulsen MK, Thomsen LP, Weitzberg E, Kamavuako EN, et al. Chronic high-dose beetroot juice supplementation improves time trial performance of well-trained cyclists in normoxia and hypoxia. Nitric Oxide. 2019 Apr 1;85:44-52.

73. Casado A, Domínguez R, Fernandes Da Silva S, Bailey SJ. Influence of Sex and Acute Beetroot Juice Supplementation on 2 KM Running Performance. Applied Sciences. 2021;11(3):977.
